# Topological optomechanical amplifier in synthetic PT -symmetry

**DOI:** 10.1515/nanoph-2021-0721

**Published:** 2022-02-14

**Authors:** Jian-Qi Zhang, Jing-Xin Liu, Hui-Lai Zhang, Zhi-Rui Gong, Shuo Zhang, Lei-Lei Yan, Shi-Lei Su, Hui Jing, Mang Feng

**Affiliations:** State Key Laboratory of Magnetic Resonance and Atomic and Molecular Physics, Wuhan Institute of Physics and Mathematics, Innovation Academy of Precision Measurement Science and Technology, Chinese Academy of Sciences, Wuhan 430071, China; School of Physics, Zhengzhou University, Zhengzhou 450001, China; National Laboratory of Solid State Microstructures, School of Physics, Nanjing University, Nanjing 210093, China; Key Laboratory of Low-Dimensional Quantum Structures and Quantum Control of Ministry of Education, Department of Physics and Synergetic Innovation Center for Quantum Effects and Applications, Hunan Normal University, Changsha 410081, China; The College of Physics and Optoelectronic Engineering, Shenzhen University, Shenzhen 518060, China; Henan Key Laboratory of Quantum Information and Cryptography, Zhengzhou, 450001, China

**Keywords:** cavity optomechanics, chirality, topological amplification

## Abstract

We propose how to achieve synthetic PT symmetry in optomechanics without using any active medium. We find that harnessing the Stokes process in such a system can lead to the emergence of exceptional point (EP), i.e., the coalescing of both the eigenvalues and the eigenvectors of the system. By encircling the EP, both nonreciprocal optical amplification and chiral mode switching can be achieved. As a result, our synthetic PT -symmetric optomechanics works as a topological optomechanical amplifier. This provides a surprisingly simplified route to realize PT -symmetric optomechanics, indicating that a wide range of EP devices can be created and utilized for various applications such as topological optical engineering and nanomechanical processing or sensing.

## 1 Introduction

Unconventional effects of exceptional points (EPs), i.e., non-Hermitian spectral degeneracies at which the eigenvalues and their eigenvectors coalesce, as revealed in recent years [1], [2], [3], [4], [5], [6], [7], [8], [9], [10], [11], [12], [13], [14], [15], [16], [17], [18], [19], [20], [21], [22], [23], [24], [25], [26], have radically changed our understanding of complex systems and led to important applications. Novel EP devices have been fabricated and utilized for realizing, e.g., ultra-sensitive metrology [27], [28], [29], [30], single-mode lasing [31], [32], [33], [34], loss-induced transparency [35, 36], and wireless power transfer [37, 38]. In particular, EP-enabled exotic topological effects have attracted intense interests [39], [40], [41], [42], [43], such as non-Hermitian skin effect [43], [44], [45], [46], topological energy transfer [47, 48], and asymmetric mode switching [49], [50], [51], [52], [53], [54], [55], [56], providing new opportunities for such a wide range of fields as synthetic photonics and topological physics [59], [60], [61]. However, due to the accumulation of dissipations in topological operations, as far as we know, topological amplifier which works as a key element in practical application has remained a challenge as topological EP devices till now.

In this work, we propose how to achieve synthetic PT symmetry and topological amplifier in optomechanics [57, 58, 62], without the need of any active medium. We find that the optomechanical Stokes processes can be harnessed to compensate the optical losses and thus realize PT symmetry in such a passive system [34, 63] without complexities, such as fabricating gain materials in active systems [1, 12, 64]. As another merit, topological optical amplifications can be realized here by simply tuning the optical modes rather than steering the acoustic modes [47] or designing materials with modulated structures [48], [49], [50], [51], [52], [53], [54], [55], [56]. Our work confirms that optomechanical systems can serve as a powerful tool to observe and utilize various topological EP effects.

In comparison with the previous works for the EP, our scheme owns significant differences as follows. First of all, in our work there exists an optical gain with a tunable center frequency as the special character of cavity optomechanics via a tunable frequency of the pump field. It is beyond the traditional gain processes, especially for the one in cavity optomechanics [34], where the center frequency of gain cannot be tuned for the certain frequency of the pump field. Secondly, different from the previous works for topological energy transfer in waveguides [48], [49], [50], [51], [52], [53], [54], [55], [56] or optomechanical phonon modes [47], which are limited by the accumulation of dissipations, our work illustrates that the dissipation accumulation can be overcome by employing the time-dependent gain with a tunable center frequency from the Stokes processes. Thirdly, contrary to the path-dependent topological dynamics with waveguides, where these fabricated optical systems lose their tunability, our optical system is time-dependent, and thus feasible to simulate the topological dynamics with different trajectories and topological properties. Finally, different from the previous work for energy transfer [47], where the effective two-level structure of phonon modes limits its application on the topological amplifier, our system benefits from the configuration of micro-toroidal resonators. This configuration leads to forward and backward transmissions taking different physical dynamics, and enables our system to work as a topological amplifier.

## Synthetic PT symmetric optomechanics

2

**Figure 1:**
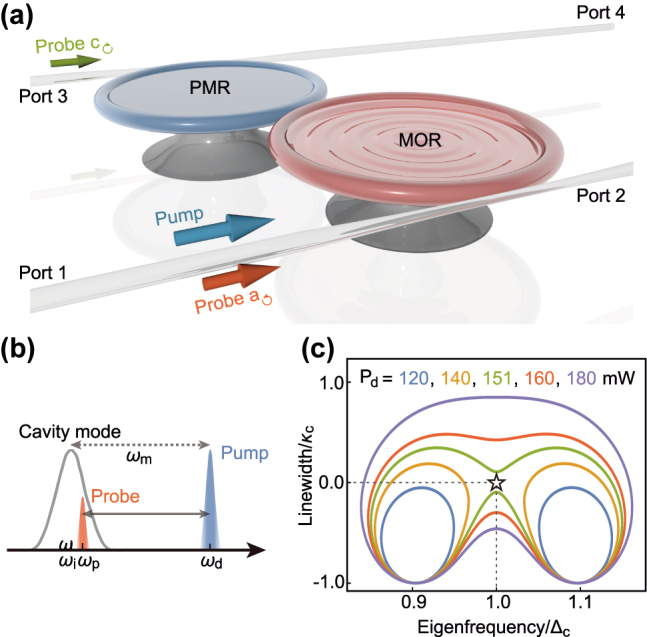
Synthetic PT symmetry in a passive optomechanical system without gain materials. (a) Two resonators, denoted by MOR and PMR, are evanescently coupled with each other and also coupled with optical fibers. A blue-detuned pump is input at the port 1 and the probe field enters from the ports 1 and 3. (b) In the propagation direction of the pump, the low-frequency probe acquires an effective gain from the pump via the Stokes process. (c) The eigenfrequency $Re\left[\omega \right]$ and its linewidth $Im\left[\omega \right]$ are the functions of the detuning Ω for different values of the pump power *P*_d_.

We start by considering a passive optomechanical system as shown in Figure 1(a), where a micro-toroidal optomechanical resonator (MOR) evanescently couples to a passive micro-toroid resonator (PMR) [34, 63]. This system can be described in the simplest level by the Hamiltonian

(1) $$H=\frac{p^{2}}{2m}+\frac{1}{2}m\omega_{m}^{2}q^{2}-\chi qa_{↺}^{†}a_{↺}+H_{\text{c}},$$

where *q* and *p* are position and momentum operators of the vibrational mode, respectively. The vibrational mode takes an effective mass *m* and an eigenfrequency *ω*_*m*_. The optical mode *a*_↺_ in MOR couples to the vibrational mode via a radiation pressure coupling *χ*. Optical mode *a*_↺_ is counter clockwise at frequency *ω*_a_, which is driven (detected) by a pump (probe) field with frequency *ω*_d_ (*ω*_p_) and amplitude $\sqrt{2\kappa_{\text{a}}}s_{\text{d}}$ $\left(\sqrt{2\kappa_{\text{a}}}s_{a_{↺}}\right)$ from input port 1, while the optical mode *c*_↻_ of PMR in clockwise at frequency *ω*_c_ is only detected by a probe field with frequency *ω*_p_ and amplitude $\sqrt{2\kappa_{\text{c}}}s_{c_{↻}}$ from input port 3. In the rotating frame at frequency *ω*_d_, the Hamiltonian for the cavities is given by

(2) $$\begin{array}{ll} H_{c}/\hbar & =\Delta_{\text{a}}a_{↺}^{†}a_{↺}+\Delta_{\text{c}}c_{↻}^{†}c_{↻}+g\left(a_{↺}^{†}c_{↻}+H.c.\right)\\ & \;+i\sqrt{2\kappa_{\text{a}}}s_{\text{d}}\left(a_{↺}^{†}-a_{↺}\right)\\ & \;+i\sqrt{2\kappa_{\text{a}}}s_{a_{↺}}\left(a_{↺}^{†}{\text{e}}^{-\text{i}\Omega t}-H.c.\right)\\ & \;+i\sqrt{2\kappa_{\text{c}}}s_{c_{↻}}\left(c_{↻}^{†}{\text{e}}^{-\text{i}\Omega t}-H.c.\right), \end{array}$$

where *a*_↺_ $\left(a_{↺}^{†}\right)$ and *c*_↻_ $\left(c_{↻}^{†}\right)$ are the annihilation (creation) operators of MOR and PMR, respectively. Ω = *ω*_p_ − *ω*_d_ (Δ_i=a, c_ = *ω*_i_ − *ω*_d_) is the detuning between the fixed probe field (cavity modes) and the tunable pump field. *g* is the evanescent coupling between MOR and PMR. $s_{\text{i}}=\sqrt{P_{\text{i}}/\hbar \omega_{\text{i}}}$ is governed by power *P*_i_ for i = p, *a*_↺_, *c*_↻_, and *κ*_a(c)_ is the decay rate for mode *a*_↺_ (*c*_↺_).

In the blue-sideband regime (Ω ≃ − *ω*_*m*_), by employing the mean-value equations for Hamiltonian (1) and eliminating the vibrational mode, we obtain the effective mean-value equations for optical modes in the frequency domain as [65, 66]

(3) $$\begin{array}{cc} -i\Omega a_{↺} & =-\left(\kappa_{\text{eff}}+i\Delta_{\text{eff}}\right)a_{↺}-igc_{↻}+\sqrt{2\kappa_{\text{a}}}s_{a_{↺}},\\ -i\Omega c_{↻} & =-\left(\kappa_{\text{c}}+i\Delta_{\text{c}}\right)c_{↻}-iga_{↺}+\sqrt{2\kappa_{\text{c}}}s_{c_{↻}}, \end{array}$$

where the effective detuning and gain are, respectively,

(4) $$\Delta_{\text{eff}}=\beta \omega_{m}\;\sin\theta /|\Omega_{m}|+\Delta_{\text{a}}-\chi q_{\text{s}}/\hbar$$

and

(5) $$\kappa_{\text{eff}}=\kappa_{\text{a}}-\beta \omega_{m}\;\cos\theta /|\Omega_{m}|$$

with Ω_*m*_ = *γ*_*m*_/2 − *i*(Ω + *ω*_*m*_), *β* = *χq*_s_/(2ℏ), e^i*θ*^ = Ω_*m*_/|Ω_*m*_|, and *q*_s_ being the steady-state position [65]. The effective detuning Δ_eff_ can be adjusted by mean photon number via the optomechanical interaction. As sketched in Figure 1(b), the Stokes photons are created at frequency *ω*_p_ ≃ *ω*_d_ − *ω*_*m*_ by emitting phonons at frequency *ω*_*m*_, resulting in an effective gain *κ*_eff_ for the probe field. This provides a natural way to reach the gain–loss balance or PT symmetry, which is fundamentally different from the previous works using active materials [31], [32], [33], tunable dissipation in passive cavities [34], and modulated structures [48], [49], [50], [51], [52], [53], [54], [55], [56].

In the adiabatic limit, the non-Hermitian Hamiltonian of optical modes *a*_↺_ and *c*_↻_ for Eq. (3) can be written in a time-dependent manner [12, 65]

(6) $$\begin{array}{cc} H_{\text{eff}}\left(t\right) & =\left(\begin{array}{cc} \Delta_{\text{eff}}\left(t\right)-i\kappa_{\text{eff}}\left(t\right) & g\\ g & \Delta_{\text{c}}-i\kappa_{\text{c}} \end{array}\right) \end{array},$$

which has the eigenmodes

(7) $$\left|\psi_{\pm }\left(t\right)\right\rangle =\left(-i\lambda \left(t\right)\pm \sqrt{1-\lambda {\left(t\right)}^{2}}\right)\left|a_{↺}\right\rangle +\left|c_{↻}\right\rangle ,$$

and the eigenvalues

(8) $$\omega =\omega_{\pm }=V\left(t\right)/2\pm g\sqrt{1-\lambda {\left(t\right)}^{2}}.$$

Here

(9) $$V\left(t\right)=\Delta_{\text{eff}}+\Delta_{\text{c}}-i\left(\kappa_{\text{eff}}+\kappa_{\text{c}}\right),$$

and

(10) $$\lambda \left(t\right)=\left[\kappa_{\text{eff}}\left(t\right)-\kappa_{\text{c}}+i\left(\Delta_{\text{eff}}\left(t\right)-\Delta_{\text{c}}\right)\right]/2g.$$

$\left|a_{↺}\right\rangle$ and $\left|c_{↻}\right\rangle$ denote optical modes *a*_↺_ and *c*_↻_, respectively.

**Figure 2:**
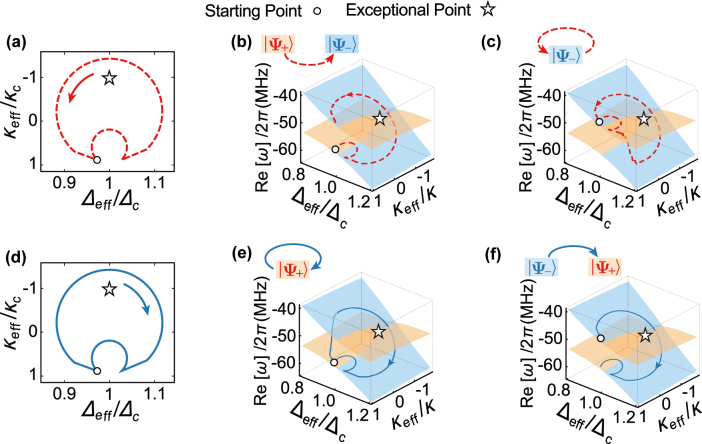
Energy dynamics $Re\left[\omega \right]$ and their trajectories with chirality follow the loops of topological operations with EP. (a and d) Loops of the topological operation, where the topological trajectories start from the initial eigenmodes plotted in the following panels. (b and e) Initial eigenmode |*ψ*_+_(*t* = 0)⟩ and (c and f) initial eigenmode |*ψ*_−_(*t* = 0)⟩ in the parameter spaces of the eigenenergy $Re\left[\omega \right]$ , effective detuning Δ_eff_ and decay rate *κ*_eff_. Arcs with arrows represent the topological operation directions in CCW (dashed red) and CW (solid blue). Here, eigenenergies for eigenmodes |*ψ*_+_⟩ and |*ψ*_−_⟩ are plotted in orange and blue. Parameters for simulations are *T* = 0.01 ms, *ω*_*m*_/2*π* = 51.8 MHz, *γ*_*m*_/2*π* = 41 kHz, *m* = 20 ng, Δ_c_ = −*ω*_*m*_, *g*/2*π* = 5 MHz, *κ*/2*π* = 5 MHz, *κ*_c_ = *κ*_a_ = *κ*, *λ* = 390 nm, *χ*/2*π* = 12 × 10^18^ℏ [8, 34, 63, 68].

The topological features of Hamiltonian (6) can be identified from the complex eigenvalues (8) versus detuning Ω with different pump power *P*_d_ as plotted in Figure 1(c). Two independent orange circles (*P*_d_ = 140 mW) are gradually melting into a big green circle (*P*_d_ = 151 mW) with the increase of the pump power *P*_d_. It is the larger pump power *P*_d_ that provides a larger effective gain and ensures the EP to be enclosed in closed circles. In addition, topological features can also be identified from the Riemann surface in Figure 2.

## 3 Topological engineering around EPs

To describe the topological dynamics around the EP, we need to acquire the effective scattering matrix of Hamiltonian (6) at first.

We assume an evolution trajectory consisting of *N* short sections, where the scattering matrix regarding the section *k* is given by

(11) $$\begin{array}{ll} U_{k}^{\text{eff}} & =\exp\left[-i\left(\delta \Delta /2-i\delta \kappa /2\right)T_{\text{0}}\right]\\ & \;*\left(\begin{array}{cc} \cos\frac{\lambda T_{\text{0}}}{2}-\eta \;\sin\frac{\lambda T_{\text{0}}}{2} & -\frac{2ig}{\lambda }\;\sin\frac{\lambda T_{\text{0}}}{2}\\ -\frac{2ig}{\lambda }\;\sin\frac{\lambda T_{\text{0}}}{2} & \cos\frac{\lambda T_{\text{0}}}{2}+\eta \sin\frac{\lambda T_{\text{0}}}{2} \end{array}\right) \end{array}$$

with

(12) $$\left\{\begin{array}{c} \lambda =\sqrt{4g^{2}+{\left(\delta \Delta -i\delta \kappa \right)}^{2}},\\ \eta =\frac{i\delta \Delta +\delta \kappa }{\lambda },\\ \delta \Delta =\Delta_{\text{eff}}\left[\left(k-1\right)T_{\text{0}}\right]-\Delta_{\text{c}},\\ \delta \kappa =\kappa_{\text{eff}}\left[\left(k-1\right)T_{\text{0}}\right]-\kappa_{\text{c}}, \end{array}\right.$$

where *T*_0_ is the evolution time for each section.

The corresponding total scattering matrix can be written as

(13) $$U=\Pi_{k=1}^{N}U_{k}^{\text{eff}},$$

which shows that the amplification of the transmission field is determined by the time-dependent net gain *δκ* via the Stokes processes, and the time-dependent evolution mode |*ψ*(*t*)⟩ can be expressed as

(14) |ψ(t)〉=U|ψ(t=0)〉,

with an initial mode |*ψ*(*t* = 0)⟩ for the time *t* = 0.

Next, to illustrate the NATs in the topological dynamics of our system, we simulate the evolution trajectories for topological operations in counter-clockwise (CCW) and clockwise (CW) following the scattering matrix (13) with *N* = 4000 in Figure 2.

Figure 2 indicates some trajectories between two eigenenergy surfaces in the parameter space as shown in Figure 2(c and e). These trajectories from the unsteady eigenmode |*ψ*_+_⟩ to the steady one |*ψ*_−_⟩ are regarding NATs. The NATs will appear when the topological operation times is longer than the coherence times of the optical modes [67]. Therefore, NATs enable topology-dependent energy transfers and own chiral properties in the dynamical encircling of the EP in the parameter space.

More specifically, our system is dominated by loss (|*κ*_eff_| < *κ*). When the high energy surface is in |*ψ*_+_⟩ (Δ_eff_ > Δ_c_), the high energy eigenmode |*ψ*_+_⟩, following the topological operations in CCW [see Figure 2(a)], will decay to its steady eigenmode |*ψ*_−_⟩ as in Figure 2(c). That is a traditional transition regarding dissipations. In contrast, when high energy surface is in |*ψ*_−_⟩ (Δ_eff_ < Δ_c_), the low energy eigenmode |*ψ*_+_⟩, following the topological operations in CW [see Figure 2(d)], will decay to the high energy eigenmode |*ψ*_−_⟩ as in Figure 2(e). That works as the counterintuitive NAT from the low energy surface to the high one. This counterintuitive transition, resulting from detuning (Δ_eff_ − Δ_c_ < 0), ensures the steady eigenmode with a higher eigenenergy as in Eq. (8).

The eigenenergy surface in Figure 2 also indicates that the EP of synthetic PT symmetric optomechanics can be induced by the effective gain from Stokes processes via radiation pressure coupling. This effective gain takes tunable frequency for the radiation pressure coupling. That is different from the traditional gain methods based on rare-earth-doped gain media [10, 28, 31], [32], [33] and stimulated Brillouin processes [17], where the effective gain is limited by the frequencies of optical modes and gain materials. Moreover, in comparison to the conventional ideas induced by the coupling strength [8, 9] and the loss [5, 36, 69], our system provides an alternative way to observe PT -symmetric breaking with the effective gain.

To illustrate the nontrivial topological properties of our system, we have to introduce the topological number. Here, we can define the topological number as vorticity *ν* in Ref. [70].

According to Ref. [70], to show the topological invariant of the topological operations, we use the invariant vorticity *ν* for eigenenergies *ω*_±_ in the complex-energy plane as

$$\nu \left(\alpha \right)=-\frac{1}{2\pi }\underset{\alpha }{\oint }{▿}_{k}\;\arg\left[\omega_{+}\left(k\right)-\omega_{-}\left(k\right)\right]\text{d}k,$$

where *α* is a closed loop in the complex-energy plane, and *k* = Ω (*k* = *P*_*d*_) is for the fixed pump field *P*_*d*_ (detuning Ω). This equation shows that the EP is (is not) enclosed in the loops of the complex-energy plane for *ν* = ±0.5 (*ν* = 0). Then we can obtain the red dash curves and the solid blue one in Figure 2, corresponding to *ν* = 0.5 and *ν* = −0.5, respectively. In addition, it is worthy to point out that the topological feature can also be observed from the linewidth and eigenenergy of the eigenmodes in Figure 1(c), where two independent orange circles (*P*_*d*_ = 140 mW) for eigenmodes will melt into a big green curve (*P*_*d*_ = 151 mW) by increasing the pump power.

## 4 Numerical simulation and discussion

In this section, we demonstrate our synthetic PT symmetric system to work as a topological amplifier with numerical simulations.

### 4.1 Topological energy transfer

To illustrate topological energy transfer and its chiral properties, we plot the trajectories for the loops of topological operations enclosing an EP in CW and CCW as in Figure 2.

When the EP is enclosed in the loops of topological operations, we assume the topological operation time *T* to be long enough in accomplishment of a single NAT. In this case, topological trajectories for different initial eigenmodes depend on the topological operation direction and own chirality. For example, only the initial mode |*ψ*_+_(*t* = 0)⟩ [|*ψ*_−_(*t* = 0)⟩] evolving along the Riemann surface in CCW (CW) can be transferred to |*ψ*_−_(*t* = *T*)⟩ ≃ |*ψ*_+_(*t* = 0)⟩ as in Figure 2(b) [|*ψ*_+_(*t* = *T*)⟩ ≃ |*ψ*_−_(*t* = 0)⟩ as in Figure 2(f)]. Otherwise, the optical mode will return to its initial mode |*ψ*_+_(*t* = *T*)⟩ ≃ |*ψ*_+_(*t* = 0)⟩ as in Figure 2(c) [|*ψ*_−_(*t* = *T*)⟩ ≃ |*ψ*_−_(*t* = 0)⟩ as in Figure 2(e)] since the NAT process blocks the eigenmodes swapping along the Riemann surface. As a result, topological energy transfer between two optical modes can be achieved, taking the feature of chirality. The above topological energy transfer and chirality can also be understood from the combination of the unsteady and steady eigenmodes, similar to the results characterized experimentally [49, 50, 54], [55], [56]. Due to this reason, this chirality can be switched off by further increasing the topological operation time *T* [65], as predicted in Ref. [12].

### 4.2 Topological amplification

The topological energy transfer mentioned above inspires us to achieve time-dependent topological optomechanical amplification for optical probe pulses with tunable topological properties by designing loops enclosing EP with large enough gain in the parameter space. Therefore, we will use probe pulses to illustrate the time-dependent topological optomechanical amplification as follows.

**Figure 3:**
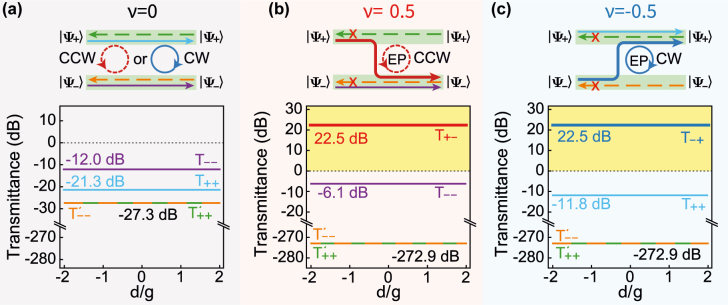
Transmission spectra *T*_*mn*_ from the input eigenmode of forward transmissions |*ψ*_*m*_(*t* = 0)⟩ to the output eigenmode |*ψ*_*n*_(*t* = *T*)⟩ with *m*, *n* = ±. Different topological dynamics is sorted via invariant vorticity *ν*. (a) Reciprocal transmission spectra *T*_*mn*_ for trival topological dynamics in CCW and CW (*ν* = 0) without the EP for the topological operation time of *T* = 0.1 μ*s* with Δ_eff_/Δ_c_ ∈ {0.9, 0.92} and *κ*_eff_/*κ*_c_ ∈ {0.15, 0.3} [65]; (b and c) Non-reciprocal transmission spectra *T*_*mn*_ for nontrival topological dynamics encircling the EP in (b) CCW (*ν* = 0.5) and (c) CW (*ν* = −0.5) with *T* = 1 μs, Δ_eff_/Δ_c_ ∈ {0.9, 1.1} and *κ*_eff_/*κ*_c_ ∈ {−1.2, 0.3} [65]. The transmission spectra are characterized with *T*_*mn*_ =⟨*ψ*_*m*_(*t* = 0)|*ψ*_n_(*t* = *T*)⟩. Detuning *δ* is the deviation frequency between the real probe pulses and the ideal ones at frequency *ω*_p_, and vorticity number *ν* is a topological invariant [65, 70, 71]. The effective parameters satisfy the approximate conditions of the theoretical derivations in supplemental materials [65]. Other parameters take the same values as in Figure 2.

To quantify the performance of time-dependent topological optomechanical amplification under the influence of the probe field frequency, we plot transmission spectra for probe pulses in Figure 3 with the scattering matrix (13) and experimentally achievable parameters [8, 34, 63, 68], which ensures the largest effective gain for simulation to be *κ*_eff_/2*π* ≃ −7.1 MHz [65] with the pump power *P*_d_ = 180 mW. Then the features of tunability, chirality, and topology can be illustrated from the transmissions in the topological dynamics with and without the EP for different values of vorticity number *ν* [65, 70, 71] as elucidated below. In the following, we assume the input probe pulses at ports 1(2) and 3(4) are in forward transmission eigenmodes |*ψ*_±_(*t* = 0)⟩ of the system [49, 50, 54], [55], [56].

When EP is not encircled by the trajectories of topological operations (*ν* = 0), the topological operations can be accomplished within the coherence time in the adiabatic limit, as mentioned by the traditional adiabatic theory [66]. This means that our system can evolve along the eigenenergy surface |*ψ*_±_⟩ and return to its initial modes |*ψ*_±_(*t* = *T*)⟩ = |*ψ*_±_(*t* = 0)⟩ without the NAT processes (see Figure S5 in [65]). In other words, no chirality exists in the transmissions as in Figure 3(a), where all the transmission spectra for topological operations in CCW and CW share the same value *T*_*mn*_ ≤ −12 dB since the loss dominates the system in this case.

On the other hand, when EP is enclosed by the trajectories of topological operations (*ν* = ±0.5), the chiral amplification of the energy can be found from the spectra difference between the forward transmissions *T*_+−_ (*T*_−+_) and *T*_−−_ (*T*_++_), which are input from ports \{1, 3\} and output at ports \{2, 4\}. Specifically, only the transmission *T*_+−_ (*T*_−+_) from the initial eigenmode |*ψ*_+_(*t* = 0)⟩ (|*ψ*_−_(*t* = 0)⟩) to the final eigenmode |*ψ*_−_(*t* = *T*)⟩ (|*ψ*_+_(*t* = *T*)⟩) in CCW (CW) can be amplified, as indicated by the solid lines in Figure 3(b and c). Otherwise, the transmission *T*_−−_ (*T*_++_) from the initial eigenmode |*ψ*_−_(*t* = 0)⟩ (|*ψ*_+_(*t* = 0)⟩) to the final eigenmode |*ψ*_−_(*t* = *T*)⟩ (|*ψ*_+_(*t* = *T*)⟩) in CCW (CW) will be suppressed by the NAT processes due to the combination of gain and loss, see the curves in Figure 2(c and e). That is to say, the chirality regarding the dynamical enclosing of an EP implies that the final eigenmodes are only determined by the directions of the topological operations in CCW and CW, and the amplification (suppression) of the initial modes depends on the directions of the topological operations, see red (dark blue) curves in Figure 3(b and c). Nevertheless, these amplifications would be less than the ones in numerical simulations for the limitation of saturation of Stokes processes [34], while the ratios of amplification of optical modes in output probe pulses will keep in a constant, since the output probe pulses are always the eigenmodes of the system.

According to the above discussion, our topological amplification (*ν* ≠ 0) results from the accumulation of the gain and loss via NAT processes. The NAT processes lead to our topological amplification beyond the adiabatic condition. Therefore, the shortest time for our topological operation is the time to finish a single NAT, i.e., constrained by both the loss and the effective gain.

### 4.3 Robustness of the topological amplification

**Figure 4:**
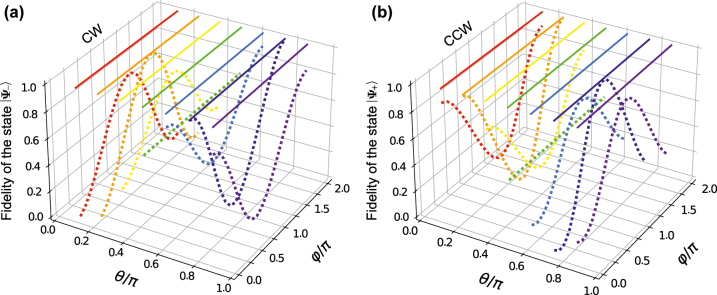
The fidelity of different topological operations in (a) CCW and (b) CW with different initial states. Here, solid lines represent topological amplification of the nontrival topological operations with an EP in (a) CCW (*ν* = 0.5) and (b) CW (*ν* = −0.5), while the dotted curves illustrate the trival topological operations without and EP in CCW and CW (*ν* = 0). Parameters in the simulations are *ω*_*m*_/2*π* = 51.8 MHz, *γ*_*m*_/2*π* = 41 kHz, *m* = 20 ng, Δ_c_ = −*ω*_*m*_, *g*/2*π* = 5 MHz, *κ*/2*π* = 5 MHz, *κ*_c_ = *κ*_a_ = *κ*, *λ* = 390 nm, *χ*/2*π* = 12 × 10^18^ℏ, and the topological operation times are *T* = 10 μs for (a and b) and *T* = 4.1 μs for (c and d). Other parameters take the same values as in Figure 2.

Now we will demonstrate the robustness of the topological amplification with different initial modes by simulating the fidelity of the final mode |*ψ*(*t* = *T*)⟩ = |*ψ*_±_⟩ with different initial mode |*ψ*(*t* = 0)⟩ = cos *θ*|*c*_↻_⟩ + e^i*ϕ*^ sin *θ*|*a*_↺_⟩ in Figure 4.

Figure 4 shows, when topological operations do not contain an EP, the initial mode is the same as the final one. It is due to the adiabatic condition. It can be identified from the oscillations of fidelities (see dotted curves). In particular, this result can be easily followed when the initial mode is in |*a*_↺_⟩. In this case, the final mode is independent of the initial angle *ϕ* and takes a constant fidelity of 0.5 (see dotted curves in green). On the other hand, when the topological operations contain an EP, the final modes must be the eigenmodes (see solid lines). We can select the eigenmodes via the evolution directions in CCW and CW (see Section 4.2). These phenomena can also be understood as the robustness original from topological properties.

### 4.4 Topological amplifier

To show our system working as a topological amplifier, we have to demonstrate the above topological optomechanical amplification owning the feature of nonreciprocity. The backward transmissions, which are input from ports \{2, 4\} and output at ports \{1, 3\}, are denoted by the dashed lines in Figure 3(b and c) with the same input probe pulses as the forward transmission. Since the backward optical modes *a*_↻_ and *c*_↺_ decouple from the forward ones *a*_↺_ and *c*_↻_, the optical mode *a*_↻_ takes a certain decay rate *κ*_a_ and an effective detuning Δ_eff_ [65]. It implies that dissipations regarding the backward transmissions only depend on the topological operation time *T*, irrelevant to the input eigenmodes and the direction of topological operations. Therefore, the backward transmissions in CCW and CW share the same value of *T*_*mn*_ = −272.9 dB for the same topological operation time, see the dashed lines in Figure 3(b and d). Here, the transmission values of *T*_*mn*_ are the ones for output pulses projected on the eigenmodes when the topological operations are finished. As probe pulses suffer from long time dissipation, the output probe pulses would be too small to be detected, and the nonreciprocal transmissions can be realized in this way.

Moreover, these final output probe pulses in Figure 3 are insensitive to the detuning *δ*. It is due to the fact that the final output probe pulses must be in one of the eigenmodes of the system, determined by the initial parameters of the pump field and selected by the direction of the topological operation.

As a result, our proposed synthetic PT symmetric optomechanics working as the topological optomechanical amplifier is feasible using current laboratory technologies [34, 63]. This optomechanical amplifier is based on the effective gain from the pump field, which opens a new way to overcome the low efficiency transmission due to the loss accumulation [49, 50, 54], [55], [56]. Compared with Ref. [34] involving a certain gain from the certain pump field, our scheme is based on a time-dependent pump field and the tunable gain enables the optical amplification with topological properties. Therefore, the accumulation of dissipations for time-dependent topological operations [56] can be overcome in this way. Moreover, our topological amplifier owns features of tunability, chirality, and topology, which are unattainable from the conventional Hermitian Hamiltonian devices [72], [73], [74], [75]. In addition, the optical gain can be enhanced by increasing the time for topological operations via the effective gain [65], and the chirality can be switched off by increasing the topological operation time. Since the final eigenmodes in our system must be in a steady eigenmode |*ψ*_−_(*t* = *T*)⟩ when all NAT processes are finished (see Figure S3 in [65]), our system offers the possibility to observe the predicted results of a long time topological dynamics around the EP [12], which overcomes the limitation of the traditional optical gain medium [1].

## 5 Conclusions

We have explored how to achieve synthetic PT symmetry in passive optomechanics [34, 63]. In such a system, we have demonstrated that topological dynamics around the EP can be selected and manipulated by a tunable blue-detuned pump field via Stokes processes from radiation pressure coupling, resulting in a topological amplifier. The proposed way of creating the effective optical gain via the blue-detuned pump field can be applied to diverse systems with similar processes, such as stimulated Brillouin scattering [76, 77], stimulated Raman scattering [78], and coupled nano-mechanical resonator array [79, 80]. Also, the synthetic PT symmetric optomechanics provides a new platform to explore time-dependent non-Hermitian dynamics [52] and topological photonics, with applications ranging from optical communications to quantum optical engineering.

## Supplementary Material

Supplementary Material Details
