# Supplementary material for: Topological optomechanical amplifier in synthetic 𝒫𝒯 -symmetry
Source: Nanophotonics. 2022 Feb 14;11(6):1149–58. doi: 10.1515/nanoph-2021-0721 (PMC11501663; doi:10.1515/nanoph-2021-0721)
Supplement: Supplementary file 1 — Supplementary Material Details [file j_nanoph-2021-0721_suppl.pdf]

Supplement Material for
"Topological optomechanical amplifier in synthetic $\mathcal{PT}$-symmetric optomechanics"

Jian-Qi Zhang, Jing-Xin Liu, Hui-Lai Zhang, Zhi-Rui Gong, Shuo Zhang, Lei-Lei Yan, Shi-Lei Su, Hui Jing, and Mang Feng

CONTENTS

S1. Hamiltonian and Mean-value equations 1

S2. Reduced mean-value equations and their effective Hamiltonian 2

S3. Solvable topological dynamics and their final modes 3
A. Reduced Hamiltonian and its eigenvectors 3
B. Hypergeometric differential equation $c(t)$ and its solutions 4
C. Evolution operator 4
1. Matrix M_1 between $[a(0), c(0)]^T$ and $[c(0), c'(0)]^T$ 5
2. Matrix M_2 between $[c(0), c'(0)]^T$ and $[c_1, c_2]^T$ 5
3. Matrix $M_3(t)$ between $[c_1, c_2]^T$ and $[c(t), c'(t)]^T$ 6
4. Matrix $M_4(t)$ between $[c(t), c'(t)]^T$ and $[a(t), c(t)]^T$ 6
5. Matrix of evolution operator from $[a(0), c(0)]^T$ to $[a(t), c(t)]^T$ 6
D. Asymptotic Analysis 6
E. Steady State of topological dynamics without chirality 7

S4. Relations of the parameters and their topological operations in the parameter spaces 8

S5. Simulations for topological dynamical steady results 8

S6. Switchable chirality of topological optomechanical amplification 10

S7. Populations for topological dynamics 11

S8. Supplementary figures for main text Figs.2 and 3 12

References 12

S1. HAMILTONIAN AND MEAN-VALUE EQUATIONS

The full Hamiltonian of the model in the main text can be described as

$$\begin{aligned}
H_{\rm tot} &= \tfrac{p^2}{2m} + \tfrac{1}{2} m\omega_{\rm m}^2 q^2 + \hbar\Delta_{\rm a} a_{\circlearrowright}^\dagger a_{\circlearrowright} + \hbar\Delta_{\rm c} c_{\circlearrowright}^\dagger c_{\circlearrowright} - \chi q a_{\circlearrowright}^\dagger a_{\circlearrowright} + \hbar g (a_{\circlearrowright}^\dagger c_{\circlearrowright} + {\rm H.c.}) \\
&\quad + i\hbar\sqrt{2\kappa_{\rm c}}\left[s_{{\rm p}-{\rm c}_{\circlearrowright}} e^{-i\Omega t} c_{\circlearrowright}^\dagger - {\rm H.c.}\right] + i\hbar\sqrt{2\kappa_{\rm a}}\left[(s_{\rm d} + s_{{\rm p}-{\rm a}_{\circlearrowright}} e^{-i\Omega t}) a_{\circlearrowright}^\dagger - {\rm H.c.}\right] + H_{\rm add} \\
H_{\rm add} &= \hbar\Delta_{\rm a} a_{\circlearrowleft}^\dagger a_{\circlearrowleft} + \hbar\Delta_{\rm c} c_{\circlearrowleft}^\dagger c_{\circlearrowleft} - \chi q a_{\circlearrowleft}^\dagger a_{\circlearrowleft} + \hbar g (a_{\circlearrowleft}^\dagger c_{\circlearrowleft} + {\rm H.c.}) \\
&\quad + i\hbar\sqrt{2\kappa_{\rm c}}\left[s_{{\rm p}-{\rm c}_{\circlearrowleft}} e^{-i\Omega t} c_{\circlearrowleft}^\dagger - {\rm H.c.}\right] + i\hbar\sqrt{2\kappa_{\rm a}}\left[s_{{\rm p}-{\rm a}_{\circlearrowleft}} e^{-i\Omega t} a_{\circlearrowleft}^\dagger - {\rm H.c.}\right].
\end{aligned} \tag{S1}$$

where q and p are corresponding to position and momentum operators of the vibrational mode, respectively. This vibrational mode takes effective mass m and eigenfrequency $\omega_{\rm m}$. The micro-toroidal optomechanical resonator(MOR) mode $a_{\circlearrowright}$ ($a_{\circlearrowleft}$) couples to the vibrational mode via a radiation pressure coupling strength χ, and interacts with the passive micro-toroid resonator (PMR) mode $c_{\circlearrowright}$ ($c_{\circlearrowleft}$) with a strength g. The optical mode $a_{\circlearrowright}$ is driven (detected) by a pump (probe) field with frequency $\omega_{\rm d}$ ($\omega_{\rm p}$) and amplitude $\sqrt{2\kappa_{\rm a}} s_{\rm d}$ ($\sqrt{2\kappa_{\rm a}} s_{\rm p}$), the rest modes i (for $i = c_{\circlearrowright}, a_{\circlearrowleft}, c_{\circlearrowleft}$) are detected by probe fields with frequency $\omega_{\rm p}$ and amplitude $\sqrt{2\kappa_{\rm c}} s_{\rm p-i}$. $\kappa_{\rm a(c)}$ is the decay rate for MOR (PMR) mode, $s_{\rm d} = \sqrt{P_{\rm d}/\hbar\omega_{\rm d}}$ is governed with the pump power $P_{\rm d}$, and Ω ($\Delta_{\rm i=a,c}$) is the detuning between the probe field (cavity modes i) and the tunable pump field.

To explore the relations between optical modes, we assume the probe field is much stronger than the quantum noise. In this situation, we can employ the Langevin equations of motion by ignoring quantum noise items of the system [D1–D3]. Then the mean-value equations for the Hamiltonian (S1) are

$$\left\{\begin{array}{lcl}\frac{d}{dt}\langle q\rangle &=& \frac{\langle p\rangle}{m},\\ \frac{d}{dt}\langle p\rangle &=& -m\omega_{\rm m}^2\langle q\rangle+\chi(\langle a_{\circlearrowright}^\dagger a_{\circlearrowright}\rangle+\langle a_{\circlearrowleft}^\dagger a_{\circlearrowleft}\rangle)-\gamma_{\rm m}\langle p\rangle,\\ \frac{d}{dt}\langle a_{\circlearrowright}\rangle &=& -(\kappa_{\rm a}+i\Delta)\langle a_{\circlearrowright}\rangle-ig\langle c_{\circlearrowright}\rangle+\sqrt{2\kappa_{\rm a}}s_{\rm d}+\sqrt{2\kappa_{\rm a}}s_{{\rm p-a}_{\circlearrowright}}e^{-i\Omega t},\\ \frac{d}{dt}\langle c_{\circlearrowleft}\rangle &=& -(\kappa_{\rm c}+i\Delta_{\rm c})\langle c_{\circlearrowleft}\rangle-ig\langle a_{\circlearrowright}\rangle+\sqrt{2\kappa_{\rm c}}s_{{\rm p-c}_{\circlearrowleft}}e^{-i\Omega t},\\ \frac{d}{dt}\langle a_{\circlearrowleft}\rangle &=& -(\kappa_{\rm a}+i\Delta)\langle a_{\circlearrowleft}\rangle-ig\langle c_{\circlearrowright}\rangle+\sqrt{2\kappa_{\rm a}}s_{{\rm p-a}_{\circlearrowleft}}e^{-i\Omega t},\\ \frac{d}{dt}\langle c_{\circlearrowright}\rangle &=& -(\kappa_{\rm c}+i\Delta_{\rm c})\langle c_{\circlearrowright}\rangle-ig\langle a_{\circlearrowleft}\rangle+\sqrt{2\kappa_{\rm c}}s_{{\rm p-c}_{\circlearrowright}}e^{-i\Omega t},\end{array}\right. \tag{S2}$$

where $\Delta=\Delta_{\rm a}-\chi\langle q\rangle/\hbar$ is the detuning, $\gamma_{\rm m}$ is the decay rate for the vibrational mode.

To have the solution to Eq. (S2), we define $O=O_{\rm s}+O_+e^{-i\Omega t}+O_-e^{i\Omega t}$, where $O=q$, p, $a_{\circlearrowleft}$, $a_{\circlearrowright}$, $c_{\circlearrowleft}$ and $c_{\circlearrowright}$, with three items $O_{\rm s}$, O_+ and O_- corresponding to the steady states at frequency $\omega_{\rm d}$, Stokes processes at frequency $\omega_{\rm p}$, and anti-Stokes processes at frequency $2\omega_{\rm d}-\omega_{\rm p}$, respectively. Then we can get the steady solutions as,

$$p_{\rm s}=0,\ q_{\rm s}=\frac{\chi|a_{\circlearrowright{\rm s}}|^2}{m\omega_{\rm m}^2},\ a_{\circlearrowright{\rm s}}=\frac{\sqrt{2\kappa_{\rm a}}s_{\rm d}}{\kappa_{\rm a}+i\Delta+\frac{g^2}{\kappa_{\rm c}+i\Delta_{\rm c}}},\ c_{\circlearrowleft{\rm s}}=\frac{-ig}{\kappa+i\Delta_{\rm c}}a_{\circlearrowright{\rm s}},\ a_{\circlearrowleft{\rm s}}=c_{\circlearrowright{\rm s}}=0, \tag{S3}$$

and the corresponding mean-value equations of Eq. (S2) for the Stokes and anti-Stokes processes can be represented as follows:

i) Equations for the Stocks processes at the frequency $\omega_{\rm p}=\omega_{\rm d}-\omega_{\rm m}$ are

$$\left\{\begin{array}{lcl}-i\Omega q_+ &=& \frac{p_+}{m},\\ -i\Omega p_+ &=& -m\omega_{\rm m}^2q_++\frac{\chi}{\hbar}a_{\circlearrowright{\rm s}}^*a_{\circlearrowright+}+\frac{\chi}{\hbar}a_{\circlearrowright{\rm s}}a_{\circlearrowright-}^*+\frac{\chi}{\hbar}a_{\circlearrowleft{\rm s}}^*a_{\circlearrowleft+}+\frac{\chi}{\hbar}a_{\circlearrowleft{\rm s}}a_{\circlearrowleft-}^*-\gamma_{\rm m}p_+,\\ -i\Omega a_{\circlearrowright+} &=& -(\kappa_{\rm a}+i\Delta)a_{\circlearrowright+}+i\frac{\chi}{\hbar}a_{\circlearrowright{\rm s}}q_+-igc_{\circlearrowleft+}+\sqrt{2\kappa_{\rm a}}s_{{\rm p-a}_{\circlearrowright}},\\ -i\Omega c_{\circlearrowleft+} &=& -(\kappa_{\rm c}+i\Delta_{\rm c})c_{\circlearrowleft+}-iga_{\circlearrowright+}+\sqrt{2\kappa_{\rm c}}s_{{\rm p-c}_{\circlearrowleft}},\\ -i\Omega a_{\circlearrowleft+} &=& -(\kappa_{\rm a}+i\Delta)a_{\circlearrowleft+}-igc_{\circlearrowright+}+\sqrt{2\kappa_{\rm a}}s_{{\rm p-a}_{\circlearrowleft}},\\ -i\Omega c_{\circlearrowright+} &=& -(\kappa_{\rm c}+i\Delta_{\rm c})c_{\circlearrowright+}-iga_{\circlearrowleft+}+\sqrt{2\kappa_{\rm a}}s_{{\rm p-c}_{\circlearrowright}}.\end{array}\right. \tag{S4}$$

ii) Equations for the anti-Stocks processes at the frequency $2\omega_{\rm d}-\omega_{\rm p}=\omega_{\rm d}+\omega_{\rm m}$ are

$$\left\{\begin{array}{lcl}i\Omega q_- &=& \frac{p_-}{m},\\ i\Omega p_- &=& -m\omega_{\rm m}^2q_-+\frac{\chi}{\hbar}a_{\circlearrowright{\rm s}}^*a_{\circlearrowright-}+\frac{\chi}{\hbar}a_{\circlearrowright{\rm s}}a_{\circlearrowright+}^*+\frac{\chi}{\hbar}a_{\circlearrowleft{\rm s}}^*a_{\circlearrowleft-}+\frac{\chi}{\hbar}a_{\circlearrowleft{\rm s}}a_{\circlearrowleft+}^*-\gamma_{\rm m}p_-,\\ i\Omega a_{\circlearrowright-} &=& -(\kappa_{\rm a}+i\Delta)a_{\circlearrowright-}+i\frac{\chi}{\hbar}a_{\circlearrowright{\rm s}}q_--igc_{\circlearrowleft-},\\ i\Omega c_{\circlearrowleft-} &=& -(\kappa_{\rm c}+i\Delta_{\rm c})c_{\circlearrowleft-}-iga_{\circlearrowright-},\\ i\Omega a_{\circlearrowleft-} &=& -(\kappa_{\rm a}+i\Delta)a_{\circlearrowleft-}-igc_{\circlearrowright-},\\ i\Omega c_{\circlearrowright-} &=& -(\kappa_{\rm c}+i\Delta_{\rm c})c_{\circlearrowright-}-iga_{\circlearrowleft-}.\end{array}\right. \tag{S5}$$

Here we assume $O_{\rm s}$ is much larger than $O_\pm$ for the fluctuations.

S2. REDUCED MEAN-VALUE EQUATIONS AND THEIR EFFECTIVE HAMILTONIAN

To reduce mean-value equations for $a_{\circlearrowright+}$ and $c_{\circlearrowleft+}$, we rewrite q_+ in Eq. (S4) with $a_{\circlearrowright+}$ as

$$q_+ \ = \ -\frac{\hbar\chi a_{\circlearrowright{\rm s}}^*[(\Delta+i\kappa_{\rm a}+\Omega)(\Delta_{\rm c}+i\kappa_{\rm c}+\Omega)-g^2]}{\hbar m[(\Delta+i\kappa_{\rm a}+\Omega)(\Delta_{\rm c}+i\kappa_{\rm c}+\Omega)-g^2](\Omega^2-\omega_{\rm m}^2+i\Omega\gamma_{\rm m})+\chi^2|a_{\circlearrowright{\rm s}}|^2(\Delta_{\rm c}+i\kappa_{\rm c}+\Omega)}a_{\circlearrowright+}. \tag{S6}$$

Then the corresponding item $i\frac{\chi a_{{\rm s}\circlearrowright}}{\hbar}q_+$ can be presented as

$$i\frac{\chi a_{\circlearrowright{\rm s}}}{\hbar}q_+ \ = \ \mu a_{\circlearrowright+}, \tag{S7}$$

where $\beta = \chi q_{\rm s}/(2\hbar)$, and $\mu = \dfrac{-2i\beta\omega_{\rm m}^2}{\Omega^2 - \omega_{\rm m}^2 + i\gamma_{\rm m}\Omega + \frac{2\beta\omega_{\rm m}^2}{\Delta+\Omega+i\kappa_{\rm a}-\frac{g^2}{\Delta_{\rm c}+\Omega+i\kappa_{\rm c}}}} \simeq \dfrac{\beta\omega_{\rm m}}{\frac{\gamma_{\rm m}}{2} - i(\Omega+\omega_{\rm m})}$ for $\Omega \simeq -\omega_{\rm m}$ [D3].

Now the Langevin equations in Eq. (S4) for optical modes $a_{\circlearrowright}$ and $c_{\circlearrowright}$ can be rewritten as [Eq. (S8) of main text Eq. (2)]

$$\begin{cases} -i\Omega a_{\circlearrowright+} &= (-\kappa_{\rm eff} - i\Delta_{\rm eff})a_{\circlearrowright+} - igc_{\circlearrowright+} + \sqrt{2\kappa_{\rm a}}s_{\rm p-a_{\circlearrowright}}, \\ -i\Omega c_{\circlearrowright+} &= (-\kappa_{\rm c} - i\Delta_{\rm c})c_{\circlearrowright+} - iga_{\circlearrowright+} + \sqrt{2\kappa_{\rm a}}s_{\rm p-c_{\circlearrowright}}, \end{cases} \tag{S8}$$

with $\kappa_{\rm eff} = \kappa_{\rm a} - {\rm Re}[\mu]$ and $\Delta_{\rm eff} = \Delta - {\rm Im}[\mu]$, and the ones for optical modes $a_{\circlearrowleft}$ and $c_{\circlearrowleft}$ are

$$\begin{cases} -i\Omega a_{\circlearrowleft+} &= -(\kappa_{\rm a} + i\Delta_{\rm eff})a_{\circlearrowleft+} - igc_{\circlearrowleft+} + \sqrt{2\kappa_{\rm a}}s_{\rm p-a_{\circlearrowleft}}, \\ -i\Omega c_{\circlearrowleft+} &= -(\kappa_{\rm c} + i\Delta_{\rm c})c_{\circlearrowleft+} - iga_{\circlearrowleft+} + \sqrt{2\kappa_{\rm a}}s_{\rm p-c_{\circlearrowleft}}. \end{cases} \tag{S9}$$

That means the radiation pressure coupling in MOR can give impacts on both the detuning and the decay rate of the optical mode $a_{\circlearrowright}$, but only affect the detuning of the optical mode $a_{\circlearrowleft}$. In other words, only the probe field in modes $a_{\circlearrowright}$ can be enhanced by the effective gain $\kappa_{\rm eff}$ via Stokes processes, which ensures our system to work as a non-reciprocal topological amplification.

Employing the method in Ref. [D4], when the linewidth of the pump field is smaller than the decay rates of the optical modes, the changes of detuning Ω, in the adiabatic limit, is in a quasi-static process. Then we can assume $\kappa_{\rm eff}(\Omega) \simeq \kappa_{\rm eff}$, and move Eq. (S8) back to the time domain as

$$\frac{d}{dt}\begin{pmatrix} a_{\circlearrowright+} \\ c_{\circlearrowright+} \end{pmatrix} = -iH_{\rm eff}\begin{pmatrix} a_{\circlearrowright+} \\ c_{\circlearrowright+} \end{pmatrix} + \begin{pmatrix} \sqrt{2\kappa_{\rm a}}s_{\rm p-a_{\circlearrowright}} \\ \sqrt{2\kappa_{\rm c}}s_{\rm p-c_{\circlearrowright}} \end{pmatrix}, \tag{S10}$$

with an effective Hamiltonian [Eq.(3) of main text]

$$H_{\rm eff} = \begin{pmatrix} \Delta_{\rm eff} - i\kappa_{\rm eff} & g \\ g & \Delta_{\rm c} - i\kappa_{\rm c} \end{pmatrix}. \tag{S11}$$

This is the Hamiltonian for numerical calculations in the main text, where the effective decay rate $\kappa_{\rm eff}$ can be achieved via optomechanically induced transparency in the blue sideband detuning [D3].

The eigenvalue for Hamiltonian (S11) is [Eq.(5) of main text]

$$\omega_{\pm} = \frac{\Delta_{\rm eff} + \Delta_{\rm c} - i(\kappa_{\rm eff} + \kappa_{\rm c})}{2} \pm \frac{1}{2}\sqrt{4g^2 + [(\Delta_{\rm eff} - \Delta_{\rm c}) + i(\kappa_{\rm c} - \kappa_{\rm eff})]^2}. \tag{S12}$$

It indicates that, in our synthetic $\mathcal{PT}$ symmetric optomechanical system, the EP occurs at $|\kappa_{\rm eff} - \kappa_{\rm c}| = 2g$ and $\Delta_{\rm eff} = \Delta_{\rm c}$, i.e., the square-root item in Eq. (S12) is zero (see main text Fig. 2).

S3. SOLVABLE TOPOLOGICAL DYNAMICS AND THEIR FINAL MODES

In this section, we first show how to derive the topological dynamical trajectories, and then we demonstrate the final mode of topological dynamics must be in the steady eigenmode when the evolution time is long enough. This steady eigenmode is determined only by initial conditions of topological dynamics.

A. Reduced Hamiltonian and its eigenvectors

The effective Hamiltonian (S11) shifted by a constant detuning $-\Delta_{\rm c}$ can be reduced to

$$H_{\rm eff-2} = \begin{pmatrix} \Delta_{\rm eff} - \Delta_{\rm c} - i\kappa_{\rm eff} & g \\ g & -i\kappa_{\rm c} \end{pmatrix}. \tag{S13}$$

It is pointed out in Ref. [D5] that the results of topological dynamics are insensitive to the deformations of the trajectories. To get the topological dynamical steady results, we assume $\Delta_{\rm eff} - \Delta_{\rm c} - i\kappa_{\rm eff} = -i(\kappa + \rho\exp[i\gamma t])$, then the effective Hamiltonian (S13) can be represented as [D5]

$$H'_{\rm eff} = \begin{pmatrix} -i(\kappa + \rho e^{i\gamma t}) & g \\ g & -i\kappa_{\rm c} \end{pmatrix}, \tag{S14}$$

where γ describes the speed of topological operation, with $\gamma > 0$ and $\gamma < 0$ corresponding to the topological operation in counter-clockwise and clockwise, respectively.

As Hamiltonian (S14) is for the two optical modes $a_{\circlearrowleft}$ and $c_{\circlearrowleft}$, its state vector can be written as $|\psi\rangle = [a(t), c(t)]^T$. Here we assume $a = a_{\circlearrowleft}$ and $c = c_{\circlearrowleft}$ for simplicity. Then, the eigenvectors for Hamiltonian (S14) are given by

$$|\psi_{\pm}\rangle = \left[-i\frac{\kappa-\kappa_c+\rho}{2g} \pm \sqrt{1-\left(\frac{\kappa-\kappa_c+\rho}{2g}\right)^2}, 1\right]^T, \tag{S15}$$

where $|\psi_+\rangle$ and $|\psi_-\rangle$ represent the eigenmodes for unsteady and steady states, respectively, since the loss dominates our system for the initial conditions [$t = 0$ for Hamiltonian (S14)].

B. Hypergeometric differential equation $c(t)$ and its solutions

According to the method in Refs. [D5, D6], we can rewrite the Schrodinger equation for Hamiltonian (S14) as

$$\begin{cases} \frac{d}{dt}a(t) + \left(\rho e^{i\gamma t} + \kappa\right) a(t) + igc(t) = 0, \\ \frac{d}{dt}c(t) + \kappa_c c(t) + iga(t) = 0. \end{cases} \tag{S16}$$

Eliminating $a(t)$ from these two equations, we find

$$\frac{d^2}{dt^2}c(t) + \left(\rho e^{ix} + \kappa_c + \kappa\right)\frac{d}{dt}c(t) + \left(\kappa_c\rho e^{ix} + g^2 + \kappa\kappa_c\right)c(t) = 0, \tag{S17}$$

where $x = \gamma t$ is a derivation with respect to the parameter x.

Defining $\xi = i\rho e^{i\gamma t}/\gamma$, we acquire the solution of $c(t)$ as

$$c(\xi) = (-\xi)^{\alpha}\left[c_1 F(p_1, p_2, \xi) + c_2 U(p_1, p_2, \xi)\right], \tag{S18}$$

where c_1 and c_2 are constants determined by the initial conditions, $p_1 = \alpha - i\kappa_c/\gamma$, $p_2 = 1 + 2\alpha - i(\kappa + \kappa_c)/\gamma$, α satisfies

$$\alpha^2 - i\alpha\left(\kappa_c + \kappa\right)/\gamma - \left(g^2 + \kappa\kappa_c\right)/\gamma^2 = 0, \tag{S19}$$

$F(p_1, p_2, \xi)$ and $U(p_1, p_2, \xi)$ are, respectively, confluent hyper-geometric functions of the first and second Kummer functions (two linear independent solutions). These two functions follow the relations as

$$\begin{cases} F(p_1, p_2, \xi) = e^{\xi}F(p_2 - p_1, p_2, e^{-\pi i}\xi), \\ U(p_1, p_2, \xi) = \frac{\Gamma(p_2 - p_1)}{\Gamma(p_2)}F(p_1, p_2, \xi)e^{i\pi p_1}\left[e^{-2i\pi p_1} - e^{-2i\pi p_2}\right] + e^{-2i\pi p_2}U(p_1, p_2, e^{-2\pi i}\xi). \end{cases} \tag{S20}$$

Defining two solutions of α in Eq. (S19) as α_1 and α_2, we rewrite the solution of $c(\xi)$ in Eq. (S18) as

$$c(\xi) = (-1)^{-\frac{i}{\gamma}(\kappa+\kappa_c)}(-\xi)^{\alpha_2}\left[c_1 U(p_1(\alpha_2), p_2(\alpha_2), \xi) + c_2 F(p_1(\alpha_2), p_2(\alpha_2), \xi)\right], \tag{S21}$$

where $(-1)^{-\frac{i}{\gamma}(\kappa+\kappa_c)}$ is a constant. This constant can be included in the constants c_1 and c_2. Note that the form of Eq. (S18) is independent from the parameter α.

C. Evolution operator

The whole evolution operator for Hamiltonian (S13) can be calculated from the following four processes:

$$[a(0), c(0)]^T \xrightarrow{M_1} [c(0), c'(0)]^T \xrightarrow{M_2} [c_1, c_2]^T \xrightarrow{M_3(t)} [c(t), c'(t)]^T \xrightarrow{M_4} [a(t), c(t)]^T. \tag{S22}$$

Now we show specifically how to get the matrix of the evolution operator.

1. Matrix M_1 between $[a(0), c(0)]^T$ and $[c(0), c'(0)]^T$

According to the initial parameters $c(0) = c(0)$, $a(0) = a(0)$, and the Schrödinger equation for $\frac{d}{dt}c(t)$ in Eq. (S16) at $t = 0$, we have the relation between $[c(0), c'(0)]^T$ and $[a(0), c(0)]^T$ as

$$\begin{pmatrix} c(0) \\ c'(0) \end{pmatrix} = M_1 \begin{pmatrix} a(0) \\ c(0) \end{pmatrix}, \tag{S23}$$

with

$$M_1 = \begin{pmatrix} 0 & 1 \\ -ig & -\kappa_c \end{pmatrix}. \tag{S24}$$

2. Matrix M_2 between $[c(0), c'(0)]^T$ and $[c_1, c_2]^T$

With the employment of Eq. (S20), the solution of $c(\xi)$ can be expressed as

$$c(t) = \left(-\frac{i\rho}{\gamma}e^{i\gamma t}\right)^{\alpha} \left[c_1 F(p_1, p_2, \frac{i\rho}{\gamma}e^{i\gamma t}) + c_2 U(p_1, p_2, \frac{i\rho}{\gamma}e^{i\gamma t})\right]. \tag{S25}$$

The first derivative of $c(t)$ is expressed as

$$\begin{aligned} \frac{d}{dt}c(t) &= \gamma\left(-\frac{i\rho}{\gamma}e^{i\gamma t}\right)^{\alpha}\left[i\alpha F(p_1, p_2, \frac{i\rho e^{i\gamma t}}{\gamma}) - \frac{\rho e^{i\gamma t}}{\gamma}\frac{p_1}{p_2}F(p_1+1, p_2+1, \frac{i\rho e^{i\gamma t}}{\gamma})\right] c_1 \\ &+ \gamma\left(-\frac{i\rho}{\gamma}e^{i\gamma t}\right)^{\alpha}\left[i\alpha U(p_1, p_2, \frac{i\rho e^{i\gamma t}}{\gamma}) + \frac{\rho e^{i\gamma t}}{\gamma}p_1 U(p_1+1, p_2+1, \frac{i\rho e^{i\gamma t}}{\gamma})\right] c_2. \end{aligned} \tag{S26}$$

For the initial values of the system $[c_1, c_2]^T$, the relations between $[c(0), c'(0)]^T$ and $[c_1, c_2]^T$ in Eqs. (S25) and (S26) is written as

$$\begin{aligned} \begin{pmatrix} c(0) \\ c'(0) \end{pmatrix} &= \gamma\left(-\frac{i\rho}{\gamma}\right)^{\alpha} M_{\mathrm{w}}(0)\begin{pmatrix} c_1 \\ c_2 \end{pmatrix}, \\ M_{\mathrm{w}}(0) &= \begin{pmatrix} F^{(0)}_{\mathrm{t=0}}/\gamma & U^{(0)}_{\mathrm{t=0}}/\gamma \\ i\alpha F^{(0)}_{\mathrm{t=0}} - \frac{\rho}{\gamma}\frac{p_1}{p_2}F^{(1)}_{\mathrm{t=0}} & i\alpha U^{(0)}_{\mathrm{t=0}} + \frac{\rho}{\gamma}p_1 U^{(1)}_{\mathrm{t=0}} \end{pmatrix}, \end{aligned} \tag{S27}$$

where the determinant $\det[M_{\mathrm{w}}(0)]$ follows

$$\det[M_{\mathrm{w}}(0)] = \left(\frac{\rho}{\gamma^2}\right)\left(\frac{i\rho}{\gamma}\right)^{-p_2}\frac{\Gamma(p_2)}{\Gamma(p_1)}e^{\frac{i\rho}{\gamma}}. \tag{S28}$$

The corresponding adjoint matrix of $M_{\mathrm{w}}(0)$ is

$$M^*_{\mathrm{w}}(0) = \begin{pmatrix} i\alpha U^{(0)}_{\mathrm{t=0}} + \frac{\rho}{\gamma}p_1 U^{(1)}_{\mathrm{t=0}} & -U^{(0)}_{\mathrm{t=0}}/\gamma \\ -i\alpha F^{(0)}_{\mathrm{t=0}} + \frac{\rho}{\gamma}\frac{p_1}{p_2}F^{(1)}_{\mathrm{t=0}} & F^{(0)}_{\mathrm{t=0}}/\gamma \end{pmatrix}, \tag{S29}$$

and the initial condition $[c_1, c_2]^T$ is given by

$$\begin{pmatrix} c_1 \\ c_2 \end{pmatrix} = \gamma^{-1}\left(-\frac{i\rho}{\gamma}\right)^{-\alpha} M^{-1}_{\mathrm{w}}(0)\begin{pmatrix} c(0) \\ c'(0) \end{pmatrix} = M_2\begin{pmatrix} c(0) \\ c'(0) \end{pmatrix} \tag{S30}$$

with

$$M_2 = \left(\frac{\gamma}{\rho}\right)\left(\frac{i\rho}{\gamma}\right)^{p_2}\left(-\frac{i\rho}{\gamma}\right)^{-\alpha}\frac{\Gamma(p_1)}{\Gamma(p_2)}e^{-\frac{i\rho}{\gamma}} \times M^*_{\mathrm{w}}(0). \tag{S31}$$

3. Matrix $M_3(t)$ between $[c_1, c_2]^T$ and $[c(t), c'(t)]^T$

According to Eqs. (S25) and (S26), we have

$$\begin{pmatrix} c(t) \\ c'(t) \end{pmatrix} = M_3(t) \begin{pmatrix} c_1 \\ c_2 \end{pmatrix} = \gamma \left(-\frac{i\rho}{\gamma} e^{i\gamma t}\right)^{\alpha} M_{\rm w}(t) \begin{pmatrix} c_1 \\ c_2 \end{pmatrix}. \tag{S32}$$

with

$$M_3(t) = \gamma \left(-\frac{i\rho}{\gamma} e^{i\gamma t}\right)^{\alpha} \begin{pmatrix} F^{(0)}/\gamma & U^{(0)}/\gamma \\ i\alpha F^{(0)} - \frac{p_1}{p_2}\frac{\rho}{\gamma} e^{i\gamma t} F^{(1)} & i\alpha U^{(0)} + p_1 \frac{\rho}{\gamma} e^{i\gamma t} U^{(1)} \end{pmatrix} = \gamma \left(-\frac{i\rho}{\gamma} e^{i\gamma t}\right)^{\alpha} M_{\omega}(t). \tag{S33}$$

4. Matrix $M_4(t)$ between $[c(t), c'(t)]^T$ and $[a(t), c(t)]^T$

With the assistance of Eq. (S16), we have

$$a(t) = i\tfrac{1}{g} c'(t) + i\tfrac{\kappa_{\rm c}}{g} c(t). \tag{S34}$$

Combined the above equation with $c(t) = c(t)$, we achieve

$$\begin{pmatrix} a(t) \\ c(t) \end{pmatrix} = M_4 \begin{pmatrix} c(t) \\ c'(t) \end{pmatrix}, \tag{S35}$$

with

$$M_4 = \begin{pmatrix} i\frac{\kappa_{\rm c}}{g} & i\frac{1}{g} \\ 1 & 0 \end{pmatrix} = M_1^{-1}. \tag{S36}$$

5. Matrix of evolution operator from $[a(0), c(0)]^T$ to $[a(t), c(t)]^T$

According to the evolution processes in Eq. (S22), the matrix for the evolutions of $[a(t), c(t)]^T$ can be calculated with matrices M_1,M_2, M_3, and M_4 as follows,

$$\begin{pmatrix} a(t) \\ c(t) \end{pmatrix} = M_4 M_3(t) M_2 M_1 \begin{pmatrix} a(0) \\ c(0) \end{pmatrix} = e^{i\alpha\gamma t} e^{-\frac{i\rho}{\gamma}} \left(\frac{i\rho}{\gamma}\right)^{p_2} \left(\frac{1}{\rho}\right) \frac{\Gamma(p_1)}{\Gamma(p_2)} \begin{pmatrix} m_{11} & m_{12} \\ m_{21} & m_{22} \end{pmatrix} \begin{pmatrix} a(0) \\ c(0) \end{pmatrix}, \tag{S37}$$

where the elements follow

$$\begin{cases} m_{11} = \frac{1}{\gamma^2}\left[(\kappa_{\rm c} + i\alpha\gamma) U^{(0)} + \rho e^{i\gamma t} p_1 U^{(1)}\right] F^{(0)}_{\rm t=0} - \frac{1}{\gamma^2}\left[(\kappa_{\rm c} + i\alpha\gamma) F^{(0)} - \rho e^{i\gamma t}\frac{p_1}{p_2} F^{(1)}\right] U^{(0)}_{\rm t=0}, \\ m_{12} = -i\frac{\kappa_{\rm c} + i\alpha\gamma}{g\gamma^2}\left[(\kappa_{\rm c} + i\alpha\gamma) U^{(0)} + \rho e^{i\gamma t} p_1 U^{(1)}\right] F^{(0)}_{\rm t=0} + i\frac{\kappa_{\rm c} + i\alpha\gamma}{g\gamma^2}\left[(\kappa_{\rm c} + i\alpha\gamma) F^{(0)} - \rho e^{i\gamma t}\frac{p_1}{p_2} F^{(1)}\right] U^{(0)}_{\rm t=0} \\ \qquad + i\frac{\rho}{g\gamma^2}\frac{p_1}{p_2}\left[(\kappa_{\rm c} + i\alpha\gamma) U^{(0)} + \rho e^{i\gamma t} p_1 U^{(1)}\right] F^{(1)}_{\rm t=0} + i\frac{\rho}{g\gamma^2} p_1 \left[(\kappa_{\rm c} + i\alpha\gamma) F^{(0)} - \rho e^{i\gamma t}\frac{p_1}{p_2} F^{(1)}\right] U^{(1)}_{\rm t=0}, \\ m_{21} = -i\frac{g}{\gamma^2}\left(U^{(0)} F^{(0)}_{\rm t=0} - F^{(0)} U^{(0)}_{\rm t=0}\right), \\ m_{22} = -\frac{1}{\gamma^2}(\kappa_{\rm c} + i\alpha\gamma) U^{(0)} F^{(0)}_{\rm t=0} + \frac{1}{\gamma^2}(\kappa_{\rm c} + i\alpha\gamma) F^{(0)} U^{(0)}_{\rm t=0} + \frac{\rho}{\gamma^2}\frac{p_1}{p_2} U^{(0)} F^{(1)}_{\rm t=0} + \frac{\rho}{\gamma^2} p_1 F^{(0)} U^{(1)}_{\rm t=0}. \end{cases} \tag{S38}$$

D. Asymptotic Analysis

With the application of Eq. (S20), we suppose the speed of the topological operations are very small ($|\gamma| \ll 1$) and the system finishes its evolution at the end time $t_{\rm end} = 2\pi/\gamma$ of the topological dynamics, yielding the following relations of the parameters,

$$\left|U^{(0)}_{\rm t=t_{end}}\right|, \left|U^{(1)}_{\rm t=t_{end}}\right| \gg \left|U^{(0)}_{\rm t=0}\right|, \left|U^{(1)}_{\rm t=0}\right|, \left|F^{(0)}_{\rm t=t_{end}}\right|, \left|F^{(1)}_{\rm t=t_{end}}\right|, \left|F^{(0)}_{\rm t=0}\right|, \left|F^{(1)}_{\rm t=0}\right|, \tag{S39}$$

and correspondingly, Eq. (S38) is reduced to

$$\begin{cases} m_{11} & \simeq \dfrac{1}{\gamma^2}\left[(\kappa_{\mathrm{c}}+i\alpha\gamma)U^{(0)}_{\mathrm{t=t_{end}}}+\rho p_1 U^{(1)}_{\mathrm{t=t_{end}}}\right]F^{(0)}_{\mathrm{t=0}}, \\ m_{12} & \simeq -\dfrac{i}{g\gamma^2}\left[(\kappa_{\mathrm{c}}+i\alpha\gamma)F^{(0)}_{\mathrm{t=0}}-\rho\dfrac{p_1}{p_2}F^{(1)}_{\mathrm{t=0}}\right]\left[(\kappa_{\mathrm{c}}+i\alpha\gamma)U^{(0)}_{\mathrm{t=t_{end}}}+\rho p_1 U^{(1)}_{\mathrm{t=t_{end}}}\right], \\ m_{21} & \simeq -i\dfrac{g}{\gamma^2}U^{(0)}_{\mathrm{t=t_{end}}}F^{(0)}_{\mathrm{t=0}}, \\ m_{22} & \simeq -\dfrac{1}{\gamma^2}\left[(\kappa_{\mathrm{c}}+i\alpha\gamma)F^{(0)}_{\mathrm{t=0}}-\rho\dfrac{p_1}{p_2}F^{(1)}_{\mathrm{t=0}}\right]U^{(0)}_{\mathrm{t=t_{end}}}, \end{cases} \tag{S40}$$

which means

$$\frac{m_{11}}{m_{21}} \simeq \frac{i}{g}\left[(\kappa_{\mathrm{c}}+i\alpha\gamma)+\rho p_1\frac{U^{(1)}_{\mathrm{t=t_{end}}}}{U^{(0)}_{\mathrm{t=t_{end}}}}\right] \simeq \frac{m_{12}}{m_{22}} \tag{S41}$$

According to Eq. (S20), in the adiabatic limit ($\gamma \to 0$), $\Gamma(p_2-p_1)/\Gamma(p_2)$ will dominate the system, implying

$$\frac{U^{(1)}_{\mathrm{t=t_{end}}}}{U^{(0)}_{\mathrm{t=t_{end}}}} \simeq -\frac{1}{p_2}\frac{F^{(1)}_{\mathrm{t=0}}}{F^{(0)}_{\mathrm{t=0}}}, \tag{S42}$$

where $F^{(1)}_{\mathrm{t=0}}/F^{(0)}_{\mathrm{t=0}}$ can be expanded to its second order as

$$\frac{F^{(1)}_{\mathrm{t=0}}}{F^{(0)}_{\mathrm{t=0}}} \approx 1-\frac{p_1-p_2}{p_2(p_2+1)}\xi+\frac{(p_1-p_2)(p_2-2p_1)}{p_2^2(p_2+1)(p_2+2)}\xi^2+O(\xi^3). \tag{S43}$$

With the assistance of $p_1=\alpha-\frac{i}{\gamma}\kappa_{\mathrm{c}}$, $p_2=1+2\alpha-\frac{i}{\gamma}(\kappa+\kappa_{\mathrm{c}})$, $\alpha=[i(\kappa_{\mathrm{a}}+\kappa_{\mathrm{c}})+\sqrt{4g^2-(\kappa_{\mathrm{a}}-\kappa_{\mathrm{c}})^2}]/(2\gamma)$, $\beta=-i/\gamma$, and $\xi_0=i\rho/\gamma$, under the condition of $\rho \ll 2g$, we can expand the ratio between two optical modes $a_{\circlearrowleft}$ and $c_{\circlearrowleft}$ to its second order as

$$\begin{aligned} \frac{a(t_{\mathrm{end}})}{c(t_{\mathrm{end}})} &= \frac{m_{11}a(0)+m_{12}c(0)}{m_{21}a(0)+m_{22}c(0)}=\frac{m_{11}}{m_{21}} \\ &\approx -\frac{i\kappa-i\kappa_{\mathrm{c}}+\sqrt{4g^2-(\kappa-\kappa_{\mathrm{c}})^2}}{2g}+\frac{\kappa-\kappa_{\mathrm{c}}-i\sqrt{4g^2-(\kappa-\kappa_{\mathrm{c}})^2}}{2g\left(\sqrt{4g^2-(\kappa_{\mathrm{c}}-\kappa_{\mathrm{c}})^2}+\gamma\right)}\rho+\frac{\left[4g^2+2i\gamma\left(\kappa-\kappa_{\mathrm{c}}-i\sqrt{4g^2-(\kappa-\kappa_{\mathrm{c}})^2}\right)\right]}{4g\left(\sqrt{4g^2-(\kappa-\kappa_{\mathrm{c}})^2}+\gamma\right)^2\left(\sqrt{4g^2-(\kappa-\kappa_{\mathrm{c}})^2}+2\gamma\right)}\rho^2+o(\rho^3) \\ &\approx -\frac{i\kappa-i\kappa_{\mathrm{c}}+\sqrt{4g^2-(\kappa-\kappa_{\mathrm{c}})^2}}{2g}+\frac{\kappa-\kappa_{\mathrm{c}}-i\sqrt{4g^2-(\kappa-\kappa_{\mathrm{c}})^2}}{2g\sqrt{4g^2-(\kappa_{\mathrm{c}}-\kappa_{\mathrm{c}})^2}}\rho+\frac{g}{\left(\sqrt{4g^2-(\kappa-\kappa_{\mathrm{c}})^2}\right)^3}\rho^2+o(\rho^3) \\ &\approx -i\frac{\kappa-\kappa_{\mathrm{c}}+\rho}{2g}-\sqrt{1-\left(\frac{\kappa-\kappa_{\mathrm{c}}+\rho}{2g}\right)^2}, \end{aligned} \tag{S44}$$

which means, at the end of topological dynamics, our system will evolve into its eigenvector

$$|\psi_-\rangle = \left[-i\frac{\kappa-\kappa_{\mathrm{c}}+\rho}{2g}-\sqrt{1-\left(\frac{\kappa-\kappa_{\mathrm{c}}+\rho}{2g}\right)^2},1\right]^T, \tag{S45}$$

corresponding to the steady eigenmode in Eq.(S15). This result is the same as the one in Ref. [D5]. In the adiabatic limit, the final ratio between the two optical modes $a_{\circlearrowleft}$ and $c_{\circlearrowleft}$ is determined by the initial conditions, but independent from the initial modes.

E. Steady State of topological dynamics without chirality

Now, we demonstrate that the steady-state of topological dynamics is independent of the directions of topological dynamics.

The Schrödinger equations for the topological dynamics in the directions of CCW and CW are

$$\begin{cases} \dfrac{da_{\mathrm{CCW}}(t)}{dt}+(\rho e^{i\gamma t}+\kappa)a_{\mathrm{CCW}}(t)+igc_{\mathrm{CCW}}(t) &= 0, \\ \dfrac{dc_{\mathrm{CCW}}(t)}{dt}+iga_{\mathrm{CCW}}(t)+\kappa_{\mathrm{c}}c_{\mathrm{CCW}}(t) &= 0. \end{cases} \tag{S46}$$

and

$$\begin{cases} \dfrac{da_{\rm CW}(t)}{dt} + (\rho e^{-i\gamma t} + \kappa) a_{\rm CW}(t) + igc_{\rm CW}(t) & = 0, \\ \dfrac{dc_{\rm CW}(t)}{dt} + iga_{\rm CW}(t) + \kappa_{\rm c} c_{\rm CW}(t) & = 0, \end{cases} \tag{S47}$$

respectively.

Taking the conjugate of Eq. (S47), we obtain

$$\begin{cases} \dfrac{da^*_{\rm CW}(t)}{dt} + (\rho e^{i\gamma t} + \kappa) a^*_{\rm CW}(t) - igc^*_{\rm CW}(t) & = 0, \\ \dfrac{dc^*_{\rm CW}(t)}{dt} - iga^*_{\rm CW}(t) + \kappa_{\rm c} c^*_{\rm CW}(t) & = 0, \end{cases} \tag{S48}$$

which could be solved by replacing $[a_{\rm CCW}(t), c_{\rm CCW}(t)]$ in Eq. (S46) with $[a^*_{\rm CW}(t), -c^*_{\rm CW}(t)]$. Then we can get the relationship between the dynamical evolutions for CCW and CW as

$$\frac{a_{\rm CCW}(t_{\rm end})}{c_{\rm CCW}(t_{\rm end})} = -\left(\frac{a_{\rm CW}(t_{\rm end})}{c_{\rm CW}(t_{\rm end})}\right)^* \tag{S49}$$

which means i) under the condition of adiabatic approximation ($\gamma \to 0$ and $t_{\rm end} \to \infty$), the final ratio between the two optical modes $a_{\circlearrowleft}$ and $c_{\circlearrowleft}$ is only determined by the initial conditions, but irrelevant to the initial modes; ii) both the evolutions in two different directions share the same output field except the phase. It results from the fact that the system approaches its steady state via the non-adiabatic transition (NAT) between the eigenmodes. The physical mechanism of the non-adiabatic transition is the hopping around an EP as discussed in Refs. [D4, D5, D7–D9]. Specially, when the initial condition of the system satisfies the large detuning, it is possible to get the eigenstates with $|\psi_+\rangle \simeq |a_{\circlearrowleft}\rangle$ for $|a_{\circlearrowleft}| \gg |c_{\circlearrowleft}|$ and $|\psi_-\rangle \simeq |c_{\circlearrowleft}\rangle$ for $|a_{\circlearrowleft}| \ll |c_{\circlearrowleft}|$.

S4. RELATIONS OF THE PARAMETERS AND THEIR TOPOLOGICAL OPERATIONS IN THE PARAMETER SPACES

To show the relationship between the experimental parameters and the effective parameters, we employ the experimentally achievable values of the detuning Ω and pump power $P_{\rm d}$ as in Fig. S1 (a,b) to calculate the effective detuning $\Delta_{\rm eff}$ and gain $\kappa_{\rm eff}$ with and without an EP enclosed in CW as in Fig. S1 (c,d). The blue solid and orange dashed curves in Fig. S1 (a-d) correspond to the topological operations with and without an EP in Figs. S1 (e) and (f), respectively.

The simulations in Fig. S1 present that the pump power $P_{\rm d}$ determines whether EP is enclosed in the loops. This is due to the fact that the largest value of the effective gain $\kappa_{\rm eff}$ is mainly determined by the pump power $P_{\rm d}$. For example, when the pump power is $P_{\rm d} = 180$ mW, the minimum value of the effective gain can reach $\kappa_{\rm eff}/2\pi = -7.134$ MHz, and the EP at the site with $\Delta_{\rm eff} = \Delta_{\rm c}$ and $\kappa_{\rm eff}/2\pi = -\kappa_{\rm c}/2\pi = -5$ MHz can be enclosed in the loops of topological operations [see blue solid curve in Fig. S1(d) and black dashed loop in Fig. S1(e)]; On the other hand, when the pump power is $P_{\rm d} = 120$ mW, the minimum value of the effective gain decreases to $\kappa_{\rm eff}/2\pi = -3.074$ MHz, and no EP is enclosed in the loops for topological operations [see orange dashed curve in Fig. S1(d) and black dashed loop in Fig. S1(f)]. Moreover, Fig. S1(d) also shows that our system is initially dominant by the loss. As a result, the final eigenmode must be in the steady eigenmode $|\psi_-\rangle$, which has been illustrated in Figs. S2 and S4(b). In addition, the reverse parameters in Fig. S1(a,b,c,d) correspond to the topological dynamics in CW.

S5. SIMULATIONS FOR TOPOLOGICAL DYNAMICAL STEADY RESULTS

In what follows, we present the topological trajectories for the system evolving to its topological steady eigenmodes with numerical calculations when the topological operation time is long enough (see Fig. S2).

As shown in the main text, when there is only one NAT with the topological operation time T, the energy transfer owns the feature of chirality, and this chirality is the combination of the steady eigenmode $|\psi_-\rangle$ and unsteady eigenmode $|\psi_+\rangle$. Therefore, when the topological operation time T is long enough to finish all the NAT processes in a topological trajectory, the unsteady eigenmode $|\psi_+\rangle$ will evolve to the steady one $|\psi_-\rangle$ via NAT due to the loss. That means final eigenmodes must be in $|\psi_-(t = T)\rangle \simeq |\psi_-(t = 0)\rangle$ as demonstrated by the blue surface in Fig. S2.

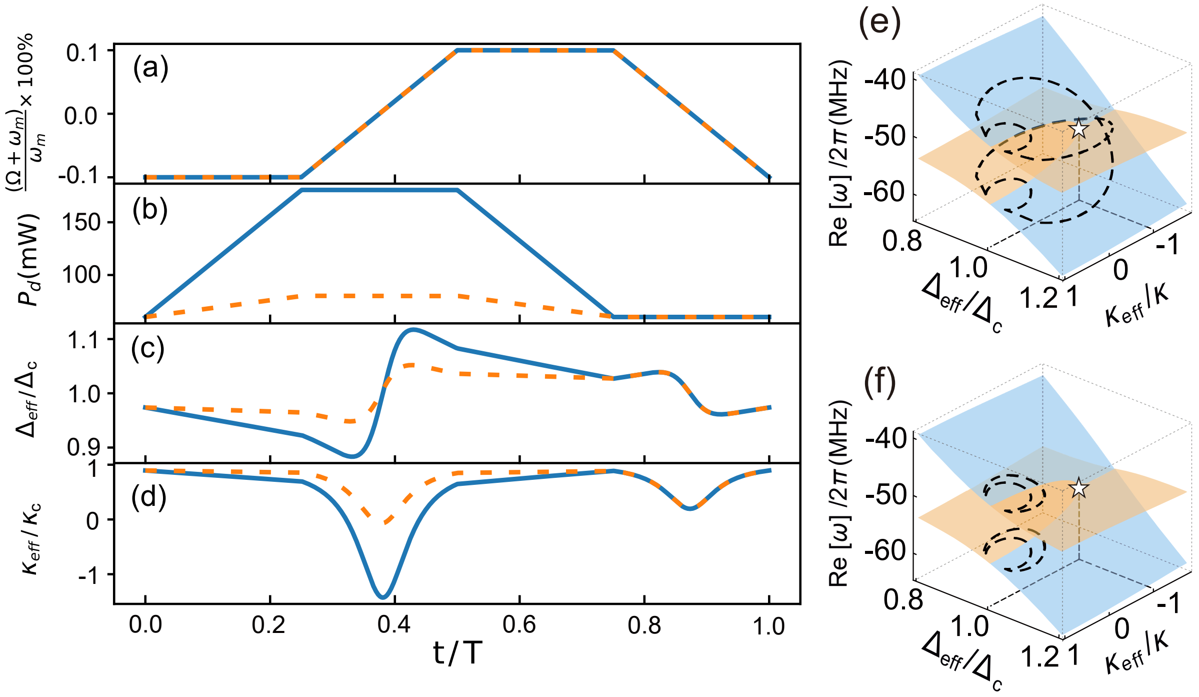

FIG. S1. Time evolution of the characteristic parameters in CCW: (a) the tunable detuning Ω, (b) the pump power P_{d}, (c) the effective detuning Δ_{eff} and (d) the effective gain κ_{eff}, where the blue solid and orange dashed curves are corresponding to dashed black loops for topological operations on Riemann surfaces (e) with an EP and (f) without an EP, respectively. Other parameters in the simulations are $\omega_{\mathrm{m}}/2\pi = 51.8$ MHz, $\Delta_{\mathrm{c}} = -\omega_{\mathrm{m}}$, $\gamma_{\mathrm{m}}/2\pi = 41$ kHz, $g/2\pi = 5$ MHz, $\kappa/2\pi = 5$ MHz, $\kappa_{\mathrm{c}} = \kappa_{\mathrm{a}} = \kappa$, $\lambda = 390$ nm, $\chi/2\pi = 12\times 10^{18}\hbar$, $m = 20$ ng. The effective detuning Δ_{eff} and gain κ_{eff} are calculated by Eqs. (S3) and(S8).

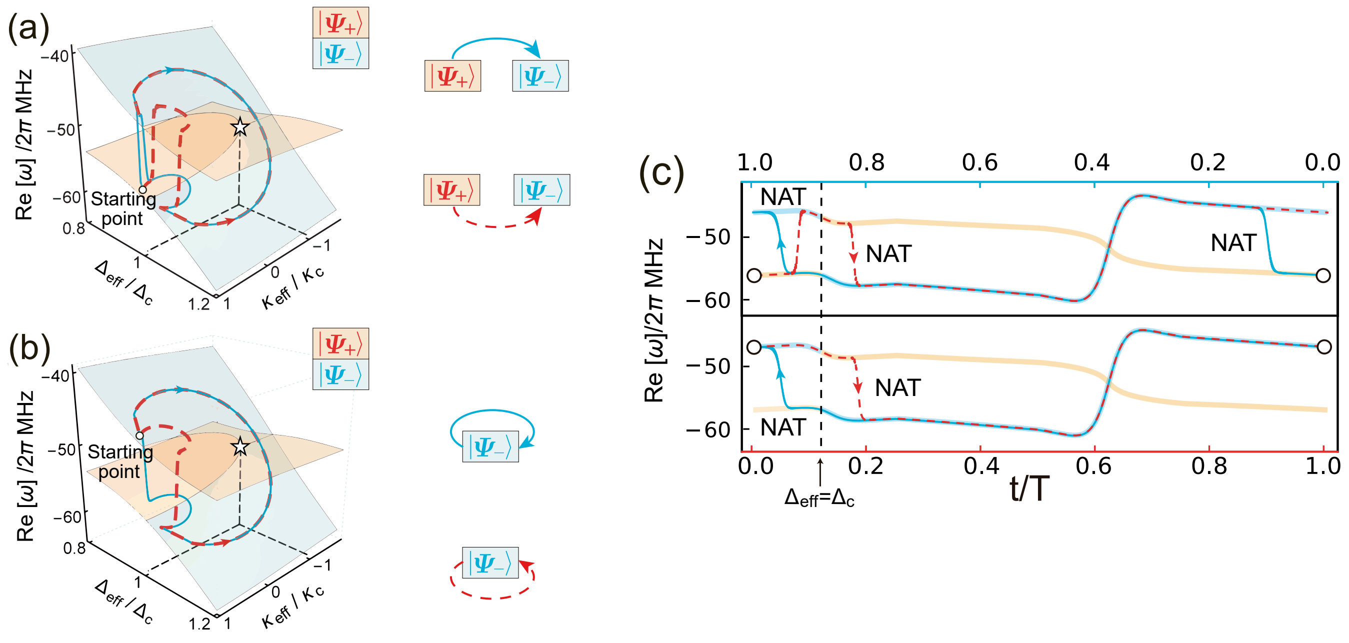

FIG. S2. Energy dynamics $\mathrm{Re}\,[\omega]$ of topological operations without chirality. Dynamics of energy $\mathrm{Re}\,[\omega]$ with (a) $|\psi_+(t=0)\rangle \simeq |a_{\circlearrowleft}\rangle$ and (b) $|\psi_-(t=0)\rangle \simeq |c_{\circlearrowleft}\rangle$ in CW (solid blue) and CCW (dashed red) as a function of time t/T on Riemann surfaces of the effective detuning Δ_{eff} and decay rate κ_{eff}. and $T = 0.06$ ms is the end time of topological operation. (c) Time evolution of energies along the direction of CW and CCW in (a,b), where eigenenergies for $|\psi_+\rangle$ and $|\psi_-\rangle$ are plotted in orange and blue. Other parameters in the simulations are the same as the ones in Fig. 2.

This final eigenmode is irrelevant to the initial modes of the system and the topological direction in CCW and CW. In this situation, no chirality can be identified from the final mode except for the starting times of the NATs as in Fig. S2(c).

S6. SWITCHABLE CHIRALITY OF TOPOLOGICAL OPTOMECHANICAL AMPLIFICATION

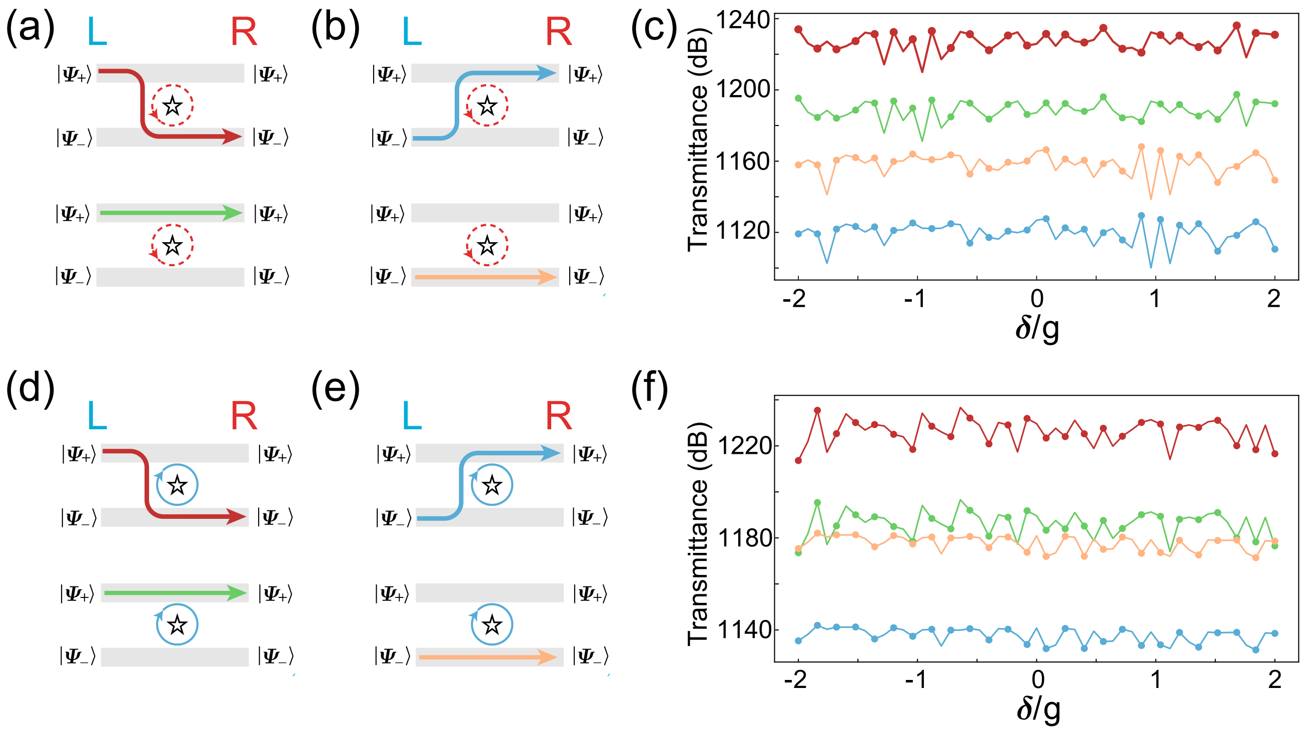

FIG. S3. Transmission spectra T_{mn} from input eigenmode $|\psi_{\mathrm{m}=\pm}(t=0)\rangle$ at ports $\{1,3\}$ to output eigenmode $|\psi_{\mathrm{n}=\pm}(t=T)\rangle$ at ports $\{2,4\}$. (a) Transmission processes of topological dynamics in CCW for initial eigenmode $|\psi_+(t=0)\rangle$; (b) Transmission processes of topological dynamics in CCW for initial eigenmode $|\psi_-(t=0)\rangle$; (c) The corresponding transmission spectra T_{mn} for (a) and (b); (d) Transmission processes of topological dynamics in CW for initial eigenmode $|\psi_+(t=0)\rangle$; (e) Transmission processes of topological dynamics in CW for initial eigenmode $|\psi_-(t=0)\rangle$; (f) The corresponding transmission spectra T_{mn} for (d) and (e). Here the end time of topological dynamics is $T=0.05$ ms. $\delta=\omega_{\mathrm{p}}'-\omega_{\mathrm{p}}$ is the detuning between the practical probe pulses at frequency ω_{p}' and the idea one at frequency ω_{p}. The effective parameters satisfy $\Delta_{\mathrm{eff}}/\Delta_{\mathrm{c}}\in\{0.9,1.1\}$ and $\kappa_{\mathrm{eff}}/\kappa_{\mathrm{c}}\in\{-1.2,0.3\}$. Other parameters in the simulation are the same as in Fig. 2.

To illustrate a topological optomechanical amplification with switchable chirality, we have to demonstrate how to achieve the chirality and how to switch off the chirality. The former has been demonstrated with the transmission spectra in the main text Fig. 3. Here we present that the chirality of topological optomechanical amplification for probe pulses can be switched off by increasing the topological operations as in Fig. S3.

From Fig. S3(c,f), we see similar T_{mn} in different topological trajectories, implying the transmission spectra of final output modes are irrelevant to the direction of topological operations. This means that no chirality can be identified from the output modes, i.e., the chirality is switched off, which is resulted from the fact that the system is approaching its steady mode $|\psi_-\rangle$ via NATs. Specifically, when the topological operation time $T=0.06$ ms is long enough for finishing all NAT processes, the unsteady eigenmode $|\psi_+\rangle$ for chirality would evolve into the steady one $|\psi_-\rangle$ via NAT due to the loss as plotted in Fig. S2. The final eigenmode $|\psi_-(t=T)\rangle\simeq|\psi_-(t=0)\rangle$ is irrelevant to the initial modes of the system and the direction of topological operations in CW and CCW. As a result, no chirality can be identified from the final modes. Although similar results have been predicted in Ref. [D5], limited by the gain materials [D10], the accumulation of dissipations makes it hard to observe. But our system provides the possibility to observe these results with the special characteristics of optomechanics for gain via the Stokes processes.

Moreover, Fig. S3(c,f) indicates that all input eigenmodes in CW and CCW can be enhanced, as the evolution time for gain is large enough to amplify all the input eigenmodes. It is worth pointing out that the transmission spectra

in Fig. S3(c,f) for input eigenmodes with different frequencies share similar values. This is because the final modes are always in the eigenmodes, which are insensitive to the deviation of the detuning δ for the probe pulses. Note that, fluctuations in transmission spectra are from the numerical accuracy of the long time evolution of topological dynamics. Similar results can also be found in Ref. [D7–D9, D11].

Although similar results have been predicted in Ref. [D5], limited by the nonlinear effects of gain materials [D10], it is impossible to be observed for the accumulation of dissipations. But our system provides a possibility to observe these results with the special character of optomechanics for gain (see the next section).

S7. POPULATIONS FOR TOPOLOGICAL DYNAMICS

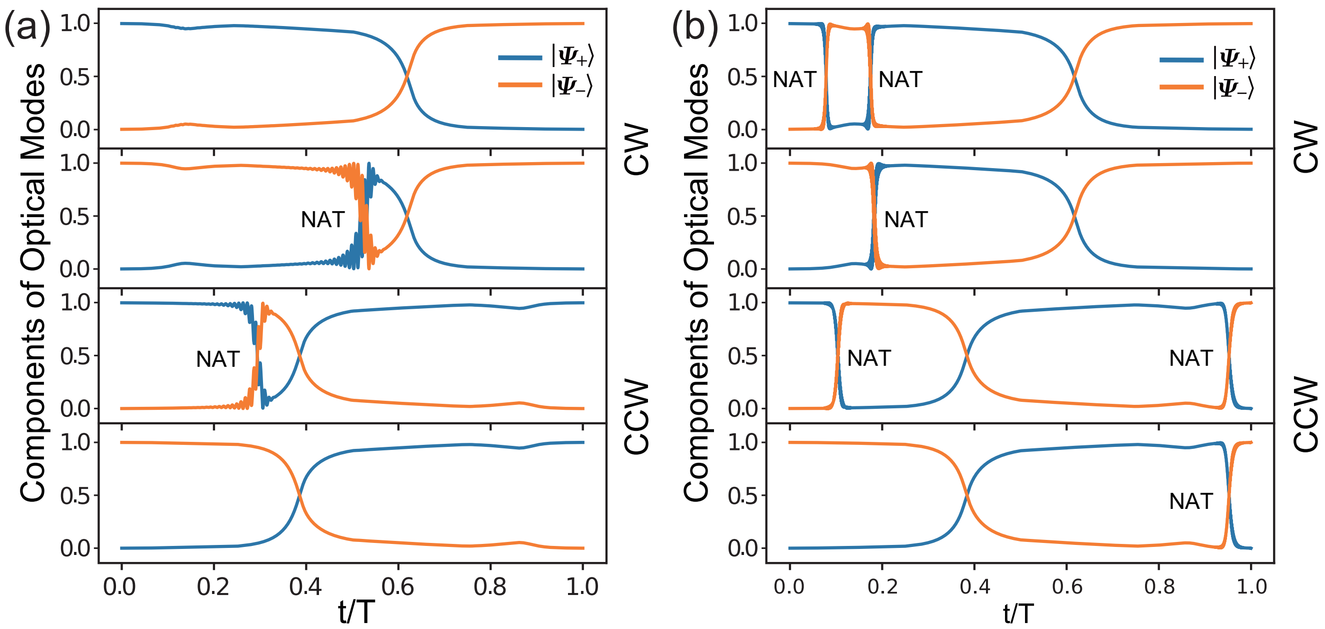

FIG. S4. Time evolution of the components of the two eigenmodes, which approximate the optical modes $|c_{\circlearrowright}\rangle \simeq |\psi_-(t=0)\rangle \simeq |\psi_-(t=T)\rangle$ (orange solid curves with population $P_{|\psi_-\rangle} \simeq |c_{\circlearrowright}|^2/(|a_{\circlearrowleft}|^2+|c_{\circlearrowright}|^2)$) and $|a_{\circlearrowleft}\rangle \simeq |\psi_+(t=0)\rangle \simeq |\psi_+(t=T)\rangle$ (blue solid curves with population $P_{|\psi_+\rangle} \simeq |a_{\circlearrowleft}|^2/(|a_{\circlearrowleft}|^2+|c_{\circlearrowright}|^2)$). Here (a) $T=0.01$ ms and (b) $T=0.1$ ms, and other parameters are the same as in Fig. 2.

To show the efficiency of the topological transfer, we define the populations of non-Hermitian eigenmodes in Fig. S4 with their biorthogonal states as:

$$P_{|\psi_\pm\rangle}(t) = \frac{\langle\psi^L_\pm(0)|\psi(t)\rangle\langle\psi(t)|\psi^R_\pm(0)\rangle}{\langle\psi^L_\pm(0)|\psi^R_\pm(0)\rangle} \tag{S50}$$

where $|\psi^R_\pm\rangle$ and $|\psi^L_\pm\rangle$ are eigenstates of H and $H^\dagger$, respectively, and satisfy $\langle\psi^L_\pm|\psi^R_\mp\rangle = 0$. Then we obtain the populations of components of eigenmodes $|\psi_\pm\rangle$ from Fig. 2 and Fig. S2 versus evolution time t, as presented in Fig. S4 (a) and (b), respectively.

Fig. S4 indicates that the switchable chirality is determined by NATs. When the topological operation time is not long enough, only one NAT occurs [see Fig. S4(a)]. In this case, only the eigenmode $|\psi_+\rangle$ ($|\psi_-\rangle$) in the direction of CW (CCW) can be transferred to the optical mode $|\psi_-\rangle$ ($|\psi_+\rangle$), following the Riemann surface. Otherwise, the single NAT will block the energy transfer from the eigenmode $|\psi_+\rangle$ ($\psi_-\rangle$) to $|\psi_-\rangle$ ($|\psi_-\rangle$). In other words, the chirality is induced by the NAT, as illustrated in the main text Fig. 2. These results are associated with the optical switch in Refs. [D8, D9].

On the other hand, with the increase of the topological operation time T, when the time T is long enough to finish all the NAT processes [see Fig. S4(b)], the unsteady eigenmode $|\psi_+\rangle$ will decay to the steady eigenmode $|\psi_-\rangle$ via NATs. Therefore, the final eigenmode must be in $|\psi_-\rangle$, as calculated in Eq. (S45), and the chirality can be switched off by the NATs.

In our system, since the probe pulses can be compensated from the pump field via Stokes processes, we can observe the long time topological steady eigenmode $|\psi_-\rangle$. This is significantly different from the previous works based on waveguides [D7–D9], where large dissipation makes it hard to observe the steady eigenmode $|\psi_-\rangle$ due to vanishing photons at the end time of topological operations.

S8. SUPPLEMENTARY FIGURES FOR MAIN TEXT FIGS.2 AND 3

In this section, we present Figs. S5 and S6 as the supplement for Figs. 2 and 3, respectively. Then we give some comments on the topological dynamics without an EP.

In Fig. S5, the fluctuation of trajectory in Fig. S5(d) is the impacts of evanescently couples and accumulation of relative phase between two optical modes in the adiabatic limit [D13]. In Fig. S6, the values of backward transmission spectra only depend on the topological operation times due to constant dissipation in the topological operations for backward transmission. The strength of the output probe pulses only depends on topological operation time. Therefore, all the backward transmission spectra of the same topological operation time take the same values.

[D1] G. S. Agarwal, and S. Huang, "Electromagnetically induced transparency in mechanical effects of light", Phys. Rev. A **81**, 041803 (2010).

[D2] S. Weis, R. Rivière, S. Delèglise, E. Gavartin, O. Arcizet, A. Schliesser, T. J. Kippenberg, "Optomechanically Induced Transparency", Science **330**, 1520 (2010).

[D3] Z. Wu, R.-H. Luo, J.-Q. Zhang, Y.-H. Wang, W. Yang, and M. Feng, "Force-induced transparency and conversion between slow and fast light in optomechanics", Phys. Rev. A **96**, 033832 (2016).

[D4] H. Xu, D. Mason, L. Jiang, and J. G. E. Harris, "Topological energy transfer in an optomechanical system with exceptional points", Nature **537** 80 (2016).

[D5] A. U. Hassan, B. Zhen, M. Soljacic, M. Khajavikhan, and D. N. Christodoulides, "Dynamically Encircling Exceptional Points: Exact Evolution and Polarization State Conversion", Phys. Rev. Lett. **118** 093002(2018).

[D6] L. J. Slater, Confluent Hypergeometric Functions (Cambridge University Press, Cambridge, England, 1960).

[D7] X.-L. Zhang, T. Jiang, and C. T. Chan, "Dynamically encircling an exceptional point in antiparity- time symmetric systems: asymmetric mode switching for symmetry-broken modes", Light Sci. Appl. **8** 88 (2019).

[D8] A. Li, J. Dong, J. Wang, Z. Cheng, J. S. Ho, D. Zhang, J. Wen, X.-L. Zhang, C. T. Chan, A. Alu, Cheng-Wei Qiu, and L. Chen, "Hamiltonian Hopping for Efficient Chiral Mode Switching in Encircling Exceptional Points", Phys. Rev. Lett **125** 187403 (2020).

[D9] J. Doppler, A. A. Mailybaev, P. Rabl, N. Moiseyev, and S. Rotter, "Dynamically encircling an exceptional point for asymmetric mode switching", Nature (London) 537, 76 (2016).

[D10] M. -A. Miri and A. Alú, "Exceptional points in optics and photonics", Science **363** 42 (2019).

[D11] Q. Liu, J. Liu, D. Zhao, and B. Wang, "On-chip experiment for chiral mode transfer without enclosing an exceptional point", Phys. Rev. A **103** 023531 (2021).

[D12] H. Shen, B. Zhen, and L. Fu, "Topological Band Theory for Non-Hermitian Hamiltonians", Phys. Rev. Lett **120** 146402 (2019).

[D13] S. N. Shevchenko, S. Ashhab, F. Nori, "Landau Zener Stückelberg interferometry", Phys. Rep. **492**, 1 (2010).

[D14] The operation times are dependent on the trajectories. Here the trajectories are different from the ones in the main text Fig.2.

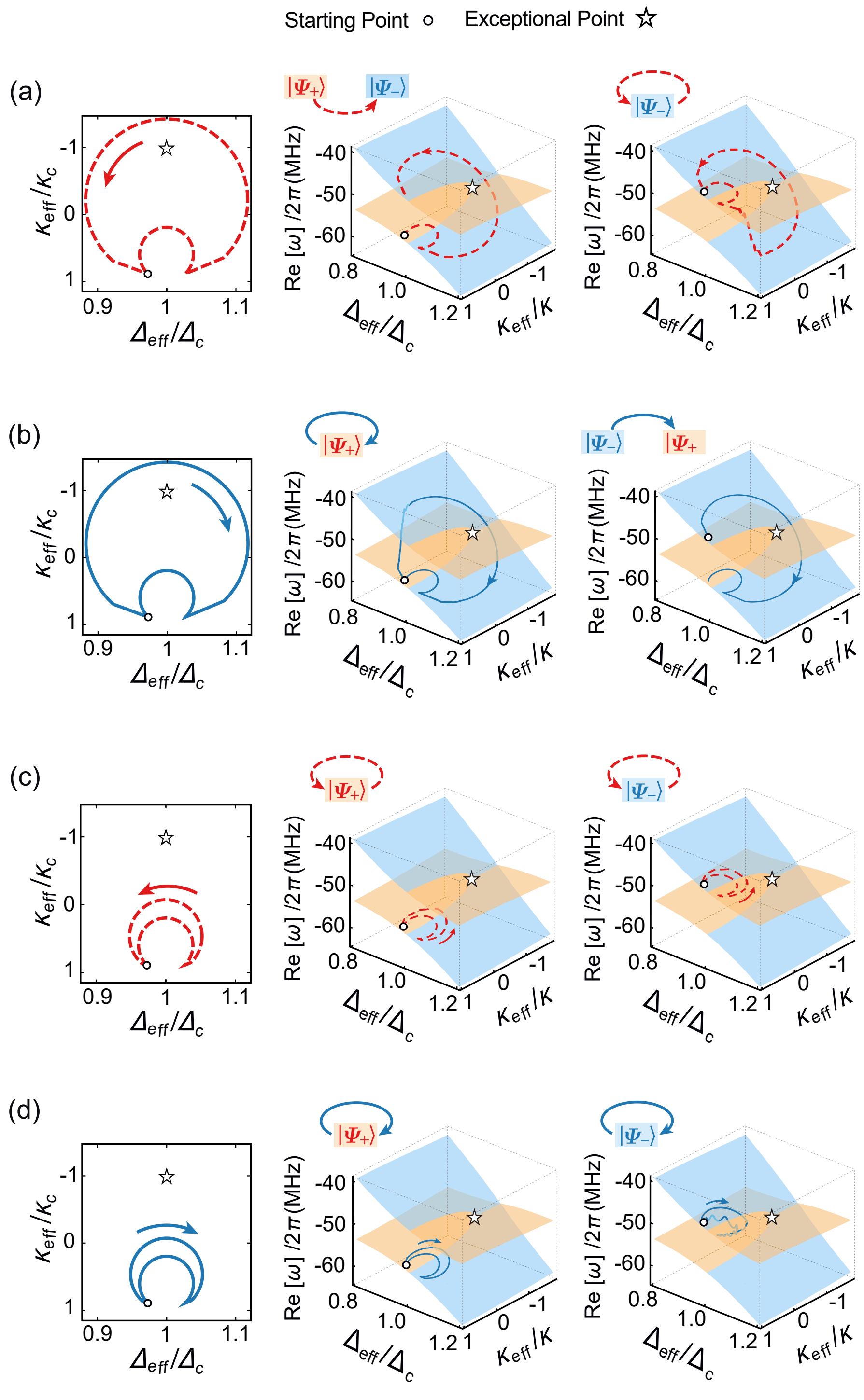

FIG. S5. Topological trajectories for energy dynamics Re $[\omega]$ following loops of topological operations (a,b) with EP and (c,d) without EP. The topological operation loop, topological trajectories starting from eigenmodes $|\psi_+(t=0)\rangle$ and $|\psi_-(t=0)\rangle$ are plotted from left to right in each diagram. Arcs with arrow indicate topological operation directions in CW (blue solid) and CCW (red dashed). The trajectories work as functions of time t/T in parameter space of eigenenergy Re $[\omega]$ versus the effective detuning $\Delta_{\rm eff}$ and decay rate $\kappa_{\rm eff}$. Here eigenenergies for $|\psi_+\rangle$ and $|\psi_-\rangle$ are plotted in orange and blue. Parameters in the simulations are $\omega_{\rm m}/2\pi = 51.8$ MHz, $\gamma_{\rm m}/2\pi = 41$ kHz, $m = 20$ ng, $\Delta_{\rm c} = -\omega_{\rm m}$, $g/2\pi = 5$ MHz, $\kappa/2\pi = 5$ MHz, $\kappa_{\rm c} = \kappa_{\rm a} = \kappa$, $\lambda = 390$ nm, $\chi/2\pi = 12 \times 10^{18}\hbar$, and the topological operation times are $T = 10$ μs for (a,b) and $T = 4.1$ μs for (c,d). These parameters are the same as main text Fig.2

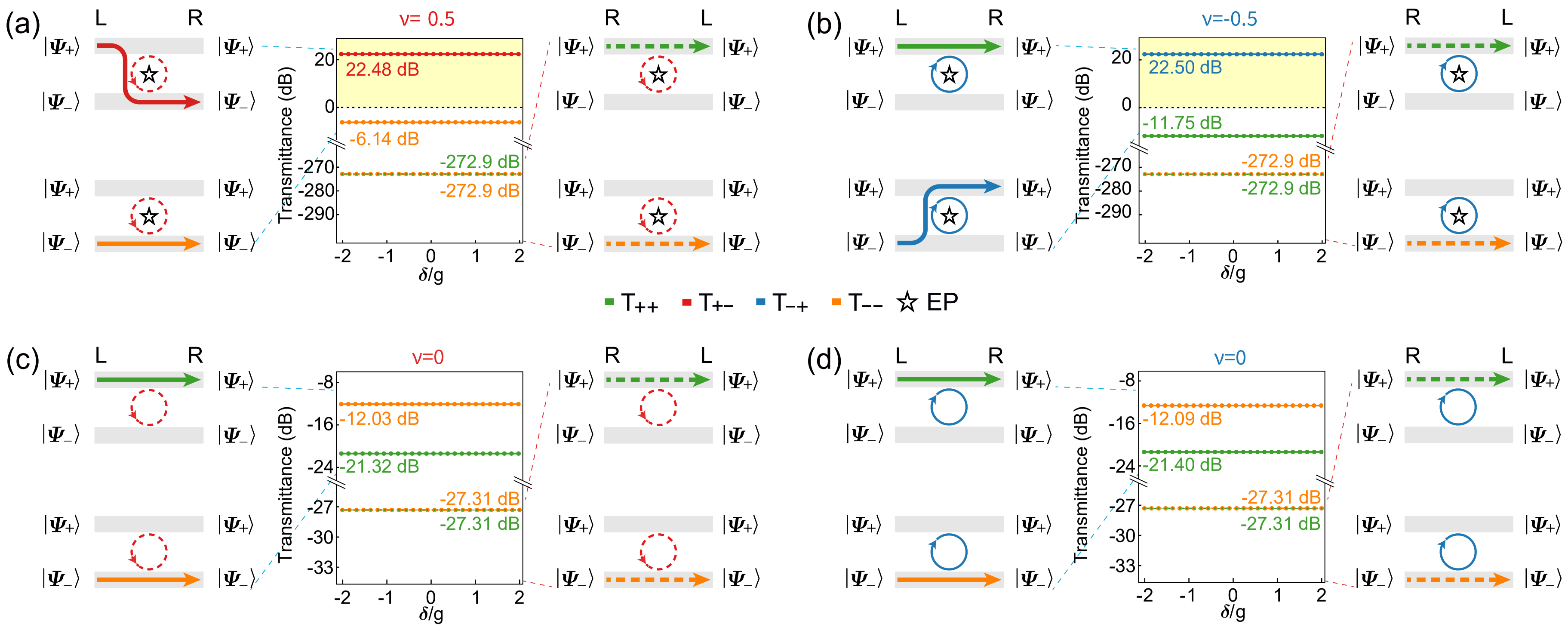

FIG. S6. Transmission spectra $T_{\rm mn}$ from input eigenmode $|\psi_{\rm m}(t=0)\rangle$ to output eigenmode $|\psi_{\rm n}(t=T)\rangle$ for $m,n=\pm$. (a,b) Transmission spectra $T_{\rm mn}$ for topological operation time $T=1$ μs [D14] with an EP, here the effective detuning follows $\Delta_{\rm eff}/\Delta_{\rm c}\in\{0.9,1.1\}$ and the effective decay satisfies $\kappa_{\rm eff}/\kappa_{\rm c}\in\{-1.2,0.3\}$; (c,d) The ones for topological operation time $T=0.1$ μs [D14] without an EP, here the effective detuning follows $\Delta_{\rm eff}/\Delta_{\rm c}\in\{0.9,0.92\}$ and the effective decay satisfies $\kappa_{\rm eff}/\kappa_{\rm c}\in\{0.15,0.3\}$). The forward transmission processes for ports {1,3} to ports {2,4}, non-reciprocal transmittance, and the backward transmission processes for ports {2,4} to ports {1,3} are plotted from left to right in each diagram. Here, we assume the speed of these topological operations shares the same value. Detuning δ is the derivation around the idea probe pulses at frequency $\omega_{\rm p}$, vorticity ν is topological invariant [D12]. The rest parameters take the same values as in Fig. 2.
